# Spatiotemporal dynamics of high and low nucleic acid-content bacterial communities in Chinese coastal seawater: assembly process, co-occurrence relationship and the ecological functions

**DOI:** 10.3389/fmicb.2023.1219655

**Published:** 2023-08-02

**Authors:** Wei Hu, Ningning Zheng, Yadi Zhang, Mark Bartlam, Yingying Wang

**Affiliations:** ^1^Key Laboratory of Pollution Processes and Environmental Criteria (Ministry of Education), Tianjin Key Laboratory of Environmental Remediation and Pollution Control, College of Environmental Science and Engineering, Nankai International Advanced Research Institute (Shenzhen Futian), Nankai University, Tianjin, China; ^2^State Key Laboratory of Medicinal Chemical Biology, College of Life Sciences, Nankai International Advanced Research Institute (Shenzhen Futian), Nankai University, Tianjin, China

**Keywords:** spatiotemporal dynamics, high and low nucleic acid-content bacteria, ecological processes, co-occurrence relationships, ecological functions

## Abstract

Studies of high nucleic acid-content (HNA) and low nucleic acid-content (LNA) bacterial communities are updating our view of their distributions and taxonomic composition. However, there are still large gaps in our knowledge of the composition, assembly processes, co-occurrence relationships and ecological functions of HNA and LNA bacterial communities. Here, using 16S rRNA gene amplicon sequencing, we investigated the spatiotemporal dynamics, assembly processes, co-occurrence relationships and ecological functions of HNA and LNA bacterial communities in the samples collected in summer and winter in Chinese coastal seas. The communities of HNA and LNA bacteria had clear spatiotemporal patterns and LNA bacteria was phylogenetically less diverse than HNA bacteria in both seasons. The distribution of HNA and LNA bacteria were significantly affected by the environmental factors and a significant seasonal-consistent distance-decay patterns were found in HNA and LNA bacteria. Furthermore, a quantitative assessment of ecological processes revealed that dispersal limitation, homogeneous selection exerted important roles in the community assembly of HNA and LNA bacteria. More importantly, we observed seasonality in the co-occurrence relationships: closer inter-taxa connections of HNA bacterial communities in winter than in summer and the opposite is true in the LNA bacterial communities. Some ecological functions, such as: phototrophy, photoautotrophy, oxygenic photoautotrophy, were different between HNA and LNA bacteria. These results provide a better understanding of spatiotemporal patterns, processes, and the ecological functions of HNA and LNA bacterial communities in Chinese coastal seawater.

## Introduction

In marine environments, microorganisms have densities of up to 10^5^–10^6^ cells/mL (Egli, 2010), with a broad diversity and a key role in the global biogeochemical cycle (Cavicchioli et al., 2003). Various studies have reported that the composition of microorganisms is regulated by complex biological and ecological factors (Chen et al., 2017; Auladell et al., 2021). Some reports have suggested that remarkable spatiotemporal patterns exist in the distribution of marine microbial taxa (Liu et al., 2020). Two different processes, determinate and stochastic processes, have been proposed to explain microbial community variations (Langenheder and Székely, 2011 Mo et al., 2018). Determinate processes involve any non-random ecological processes, including environmental filtering and diverse biological interactions such as competition, promotion, mutual benefit and predation (Stegen et al., 2012). In contrast, stochastic processes include typically random events, such as probabilistic dispersal, ecological drift, and random birth-death events (Stegen et al., 2013; Zhou, 2017). Additionally, the different aspects of environmental selection and stochasticity were discerned by researchers using a framework that integrates a null model and phylogenetic information (Stegen et al., 2013; Ning et al., 2020). Different ecological processes have been documented to exert different roles among organismal community assembly in the different ecosystems, including bacteria in lake (Logares et al., 2018), the archaea in coastal sediments (Liu et al., 2020), and microbial eukaryotes in seawater (Wu et al., 2018).

Flow cytometry (FCM) is a powerful tool that has been used to explore bacterial communities (Wang et al., 2009; Huete-Stauffer and Morán, 2012). Several groups of marine bacteria were identified based on nucleic acid staining using FCM (Li et al., 1995; Gasol et al., 1999), and were divided into low nucleic acid-content (LNA) bacteria and high nucleic acid-content (HNA) bacteria (Lebaron et al., 2001). LNA bacteria, ranging from 20 to 90% of the total bacterial community in marine environments, varies depending on the environmental conditions and ecosystem productivity (Lebaron et al., 2001; Andrade et al., 2007; Schattenhofer et al., 2011). Early work showed that LNA bacteria were considered to be dead, inactive and dormant microbes (Gasol et al., 1999), which have lower cell-specific metabolic activity than HNA bacteria (Longnecker et al., 2005). Subsequent work found that LNA bacteria have normal physiological characteristics, such as small cell size, small genomes, oligotrophic growth capacity and nutrient uptake (Hu et al., 2022). Based on these physiological characteristics, researchers found that 0.45 μm membrane filters can effectively separate HNA and LNA bacteria (Wang et al., 2009), and that filtration through micropore membrane filters (e.g., 0.45, 0.2 and 0.1 μm) has widespread applications for separating and enriching LNA bacteria (Song et al., 2019b). Meanwhile, an isolation method for LNA pure cultures through 0.45 μm filtration and oligotrophic medium was established by the authors (Wang et al., 2009).

To date, the majority of studies related to HNA and LNA bacteria have focused largely on their distribution, activities and compositions in the environment (Joux et al., 2005; Longnecker et al., 2005; Vila-Costa et al., 2012; Liu et al., 2016). Previous studies have reported that the distribution of LNA and HNA bacteria is strongly regulated by environmental variables (Nishimura et al., 2005; Liu et al., 2016, 2017; Eswaran and Khandeparker, 2021) and changes seasonally (Eswaran and Khandeparker, 2021). Researchers have tried to determine the phylogenetic composition of HNA and LNA bacteria, but have reached different conclusions. Some have postulated that HNA and LNA bacteria are two distinct microbial communities with their own independent physiological features, with little or even no interaction occurring between HNA and LNA bacteria (Bernard et al., 2000). In contrast, others have suggested that LNA and HNA bacterial groups have exclusive bacterial fractions and the same bacterial species (Vila-Costa et al., 2012). In terms of ecological function, however, LNA and HNA bacteria have distinct metabolic and ecological functions (Song et al., 2019b). Hence, there is clearly much to be learned about this key aspect of the structure of HNA and LNA bacterial communities.

In this study, we examined the geographical patterns of HNA and LNA bacterial communities in four Chinese coastal seas in both summer and winter. The composition and relationship of the HNA and LNA bacterial communities were analyzed. Furthermore, we provide a quantitative assessment of the ecological processes governing HNA and LNA bacterial community assembly and examined the co-occurrence patterns and the ecological functions of HNA and LNA bacteria under the spatiotemporal dynamics.

## Materials and methods

### Sample collections

A total of 76 samples (26 samples collected in summer and 50 samples collected in winter) were collected from four seas off the coast of China, i.e., the South China Sea (SCSS), the East China Sea (ECS), the Yellow Sea (YS), and the Bohai Sea (BHS), as shown in Supplementary Figure S1. The sampling map was generated using ODV software (Zhang et al., 2021). Surface seawater samples (5 L each) were collected at each site. All samples were stored at 4°C during transportation.

### Physicochemical parameters

The water temperature (T), pH and salinity of each sample was measured *in situ* using a Portable Thermol Multifunctional Meter (ORION 520 M-01A). Total organic carbon (TOC), total carbon (TC) and total nitrogen (TN) were measured using the high-temperature (680°C) catalytic oxidation method on a TOC analyzer (multiN/C3100, Analytikjena, Germany). Twenty milliliters samples were pre-filtered through a 0.45 μm filter membrane (PVDF, Millipore, United States) to remove the microbes and solid particles before following nutrient (TOC, TC, TN) measurement. Ammonia (NH_4_-N), total phosphorus (TP), nitrites (NO_2_-N) and nitrates (NO_3_-N) were measured using a multiparameter water quality analyzer (DR3900, Hach Company, Loveland, CO, United States). Supplementary Table S1 shows the physicochemical parameters of these samples.

### Flow cytometry, fluorescent staining and filtration protocol

Total bacterial cell counts in all water samples were measured using a combination of a fluorescent stain (SYBR Green I, Life Technologies, United States; final concentration 1:10000) (Song et al., 2019b) and FCM (BD Accuri™C6 Plus, United States) equipped with a 50 mW laser emitting at a fixed 488 nm wavelength. Briefly, 500 μL of water samples in triplicate were pipetted into 1.5 mL sterilized Eppendorf tubes and incubated in a heating block at 37°C for 5 min. Then 5 μL of fluorescent stain was added into each prewarmed water sample. After 10 min of incubation in the dark at 37°C, 50 μL of each prepared sample was measured on a flow cytometer at a fast flow rate of 66 μL/min. Fluorescent signals were collected at FITC-A = 533 ± 30 nm and PerCP-A = 670 nm, and the FSC light signal was collected as well. Samples with relatively high cell concentrations were diluted using ultrapure water to avoid the real-time counting of the flow cytometer that exceeded 1,000 cells/μL. All samples were analyzed with the exact same protocol and the same FCM gate was used for all samples to select for HNA and LNA bacteria to ensure comparability. Filtration volumes were adjusted (between 100–4,000 mL) to gather an approximately equalized cell number (~10^8^ cells in the present study) on each membrane filter (Song et al., 2019b). Separately, a two-step filtration was performed to obtain HNA and LNA bacteria. First, each water sample was filtered through a 0.45 μm membrane filter (“0.45 μm captured bacteria”) (Durapore^®^, Merck Millipore, United States) to capture the HNA bacteria and the resulting filtrate was subsequently filtered again on a 0.1 μm (“0.45 μm filterable bacteria”) (Durapore^®^, Merck Millipore, United States) membrane filter (Durapore^®^, Merck Millipore, USA) to capture the LNA bacteria (Song et al., 2019b). After these filtrations, all membrane filters were directly processed for DNA extraction.

### DNA extraction, 16S rRNA amplicon sequencing, and sequence processing

Total DNA was extracted with the Power Water DNA isolation kit (QIAGEN GmbH, Germany). The DNA yield was quantified with a Qubit® 2.0 Fluorometer (Invitrogen, Thermo Fisher Scientific Inc., United States) using a Qubit® dsDNA BR Assay kit (Invitrogen, Life technologies, Thermo Fisher Scientific Inc., USA). DNA library sequencing was performed on the Illumina HiseqTM 2,500 by Gene Denovo Biotechnology Co., Ltd. (Guangzhou, China). Primer pairs were chosen to amplify the V3-V4 hypervariable region of bacterial 16S rRNA genes (Logares et al., 2013). The 16S rDNA target region of the ribosomal RNA gene were amplified by PCR (95°C for 5 min, followed by 30 cycles at 95°C for 1 min, 60°C for 1 min, and 72°C for 1 min and a final extension at 72°C for 7 min) using primers. 50 μL mixture containing 10 μL of 5 × Q5@ Reaction Buffer, 10 μL of 5 × Q5@ High GC Enhancer, 1.5 μL of 2.5 mM dNTPs, 1.5 μL of each primer (10 μM), 0.2 μL of Q5@ High-Fidelity DNA Polymerase, and 50 ng of template DNA. Related PCR reagents were from New England Biolabs, United States. Raw data were processed and analyzed using FASTP (version 0.18.0) to remove low-quality reads, briefly, the reads containing more than 10% of unknown nucleotides (N) and containing less than 50% of bases with quality (*Q*-value) > 20 were removed. Paired end clean reads were merged as raw tags using FLASH (version 1.2.11) with a minimum overlap of 10 bp and mismatch error rates of 2%. Sequences were quality controlled with the following settings: maximum number of consecutive low-quality base =3, minimum of continuous high-quality base = 75% of total read length (Bokulich et al., 2013). The clean tags were clustered into operational taxonomic units (OTUs) of ≥ 97% similarity using UPARSE (version 9.2.64) pipeline (Edgar, 2013). All chimeric tags were removed using UCHIME algorithm and finally obtained effective tags for further analysis (Knight, 2011). Functional Annotation of Prokaryotic Taxa (FAPROTAX) was carried out to annotate bacteria functions (Louca et al., 2016). In this study, 152 water samples in both seasons were processed in the sequencing analysis. And a proportion of the winter samples (28 samples of LNA bacteria and 21 samples of HNA bacteria) were eliminated because the DNA concentration was too low to meet the requirements for subsequent sequencing analysis.

### Statistical analyses

#### Null model

The framework to quantitatively infer community assembly mechanisms by phylogenetic bin-based null model analysis (iCAMP) was developed by Ning et al. (2020). To quantify various ecological processes, the observed taxa are first divided into different groups (‘bins’) based on their phylogenetic relationships. Then, the process governing each bin is identified based on null model analysis of the phylogenetic diversity using the beta Net Relatedness Index (βNRI), and taxonomic β-diversities using the modified Raup-Crick metric (RC). For each bin, the fraction of pairwise comparisons with βNRI < −1.96 is considered as the percentages of homogeneous selection, whereas those with βNRI > +1.96 as the percentages of heterogeneous selection. Next, the taxonomic diversity metric RC is used to partition the remaining pairwise comparisons with |βNRI| ≤ 1.96. The fraction of pairwise comparisons with RC < −0.95 is treated as the percentages of homogenizing dispersal, while those with RC > +0.95 as dispersal limitation. The remains with |βNRI| ≤ 1.96 and |RC| ≤ 0.95 represent the percentages of drift.

#### Network analysis

Co-occurrence networks were constructed using the igraph, Hmisc and qvalue libraries in R (Ma et al., 2020). To reduce complexity, Spearman’s rank coefficients (ρ) between all 97%-cutoff OTUs with occurrence in at least 20% of samples and at least 60 reads were calculated pairwise using the R package Hmisc (Hu et al., 2017). The pairwise Spearman’s correlations between OTUs were calculated, with a correlation coefficient > |0.6| and a *p*-value <0.01 being considered a valid relationship (Hu et al., 2017). Network visualization was conducted using Gephi.^1^ The network-level and node-level topological features of each network were calculated.

#### Other statistical analysis

Alpha diversity, including the Shannon and Simpson index, were calculated in QIIME (version 1.9.1) (Caparoso et al., 2010). NMDS (non-metric multidimensional scaling) of unweighted unifrac was carried out to visualize the differences in compositions and the functions between HNA and LNA bacteria in two seasons. The PERMANOVA test was generated using the Vegan package in R (version 2.5.3) (Wu and Huang, 2019) to test the statistical significance of the differences and the variation explained by different groups. Redundancy analysis (RDA) was performed to explore the constraints placed on bacterial functions by environment physicochemical factors. The geosphere library was used to plot the pairwise geographic distances between samples calculated from the latitude and longitude coordinates and the pairwise Bray-curtis dissimilarities using the ggplot2 package in R (Liu et al., 2020). The linear regression models were used to explore the relationships between Bray-curtis dissimilarities and geographic distances. The Mantel and partial Mantel test analysis were used to explore the effects of environmental factors on LNA and HNA bacterial communities in two seasons.

#### Accession number

All sequencing data from this paper were deposited to the National Center for Biotechnology Information (NCBI) with accession number PRJNA876752.

## Results

### FCM analysis of HNA and LNA bacteria

**Figure 1:**
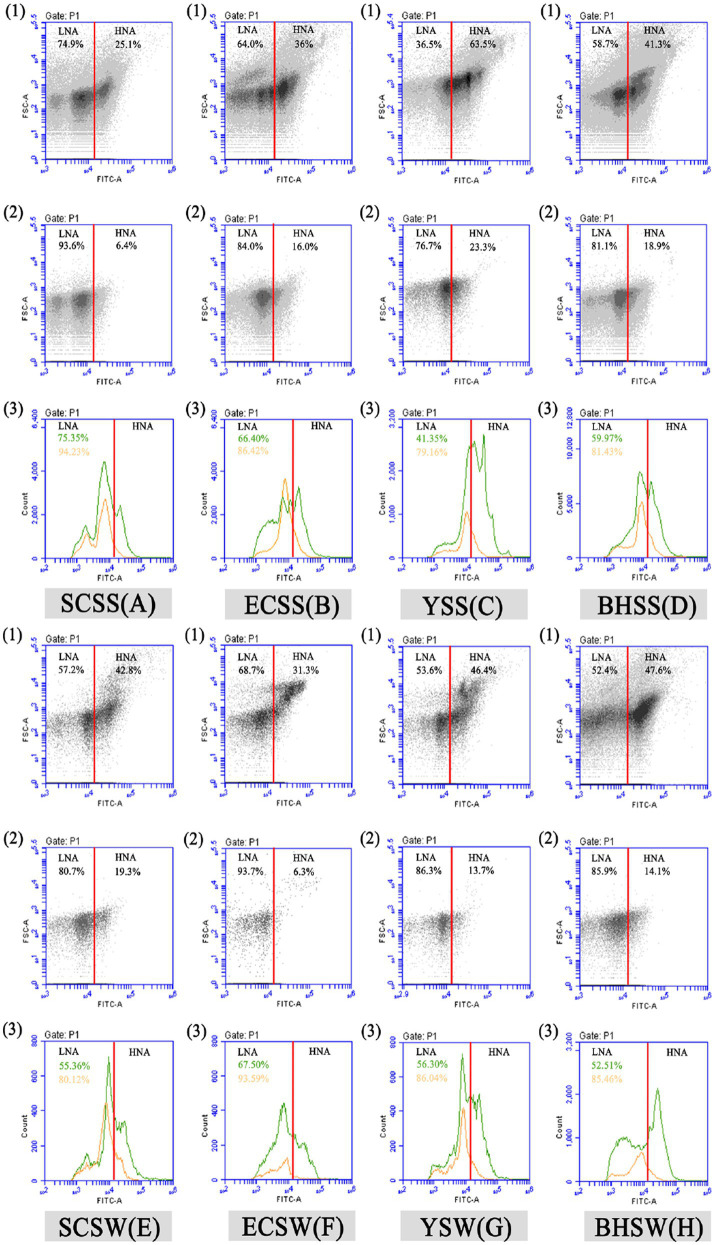
Flow cytometric fingerprint of five seawater samples. Dot plots (1 and 2) represent bacterial groups before (1) and after (2) 0.45 μm filtration in each lake sample after SYBR Green I staining. Two bacterial groups were distinguished, namely, “LNA” and “HNA,” respectively. Two peak diagrams (3) show the cell count of LNA/HNA by flow cytometric FITC-A (nucleic acid content) fluorescent and FSC (cell size) light signals before (green) and after (orange) 0.45 μm filtration in each sample, respectively, and the percentage of LNA bacteria before (green) and after (orange) 0.45 μm filtration in each sample was shown in Figure 1 (3). SCSS: samples from South China Sea in summer; SCSW: samples from South China Sea in winter; ECSS: samples from East China Sea in summer; ECSW: samples from East China Sea in winter; YSS: samples from Yellow Sea in summer; YSW: samples from Yellow Sea in winter; BHSS: samples from Bohai Sea in summer; BHSW: samples from Bohai Sea in winter.

After excluding the abiotic background through FCM gating (Supplementary Figure S2), an average total bacterial concentration of ~10^6^ cells/mL was detected, of which LNA bacteria generally accounts for >50% in terms of cell concentration (Supplementary Figure S3D). As the previous study reported (Song et al., 2019a,b), the result of this study did not exhibit a threshold between these bacterial clusters from fluorescent signal channels (FL1 versus FL3). However, when combined with the FSC light signal (cell size), a threshold of ~10^4^ on both FSC-A light signal and FITC-A fluorescent signal was used to separate different bacterial groups on the basis of bacterial size and nucleic acid content (Figure 1). The threshold on the green fluorescence channel (FITC-A) was set at 1,000 to exclude instrument noise (Proctor et al., 2018; Song et al., 2019b).The cell count of HNA and LNA bacteria in summer were both higher than that in winter (Supplementary Figures S3A,C).

The results of correlation analysis showed that HNA and LNA bacteria count were significantly positively correlated with NO_2_-N (*p* < 0.05), pH (*p* < 0.05), T (*p* < 0.05), respectively and significantly negatively correlated with salinity (*p* < 0.05). HNA bacteria percentage was significantly negatively correlated with NH_4_-N (*p* < 0.05), TN (*p* < 0.05), TC (*p* < 0.05), respectively and significantly positively correlated with T (*p* < 0.05). LNA bacteria percentage was significantly positively correlated with NH_4_-N (*p* < 0.05), TN (*p* < 0.05), TC (*p* < 0.05), respectively and significantly negatively correlated with T (*p* < 0.05). The HNA:LNA ratio was significantly negatively correlated with NH_4_-N (*p* < 0.05), TN (*p* < 0.05), TC (*p* < 0.05), respectively and significantly positively correlated with T (*p* < 0.05) (Supplementary Figure S4). Those results indicated that the distributions of LNA and HNA bacteria were strongly regulated by environmental variables. The median values of flow cytometric FSC (cell size) signal in all samples were evaluated for LNA and HNA bacteria, respectively (Supplementary Figure S5A). The results of correlation analysis showed that the FSC median values of HNA and LNA bacteria were significantly negatively correlated with T (for HNA bacteria: *r* = −0.326, *p* < 0.01; for LNA bacteria: *r* = −0.499, *p* < 0.01), pH (for HNA bacteria: *r* = −0.384, *p* < 0.01; for LNA bacteria: *r* = −0.395, *p* < 0.01) and positively correlated with TC (for HNA bacteria: *r* = 0.443, *p* < 0.01; for LNA bacteria: *r* = 0.804, *p* < 0.01), TN (for HNA bacteria: *r* = 0.492, *p* < 0.01; for LNA bacteria: *r* = 0.749, *p* < 0.01), NH_4_-N (for HNA bacteria: *r* = 0.297, *p* < 0.01; for LNA bacteria: *r* = 0.542, *p* < 0.01) and NO_3_-N (for HNA bacteria: *r* = 0.355, *p* < 0.01); for LNA bacteria: (*r* = 0.272, *p* < 0.05; Supplementary Figure S5). The results of correlation analysis showed that the bacterial cell size can gradually change with environmental conditions.

### Phylogenetic diversity of HNA and LNA bacteria

**Figure 2:**
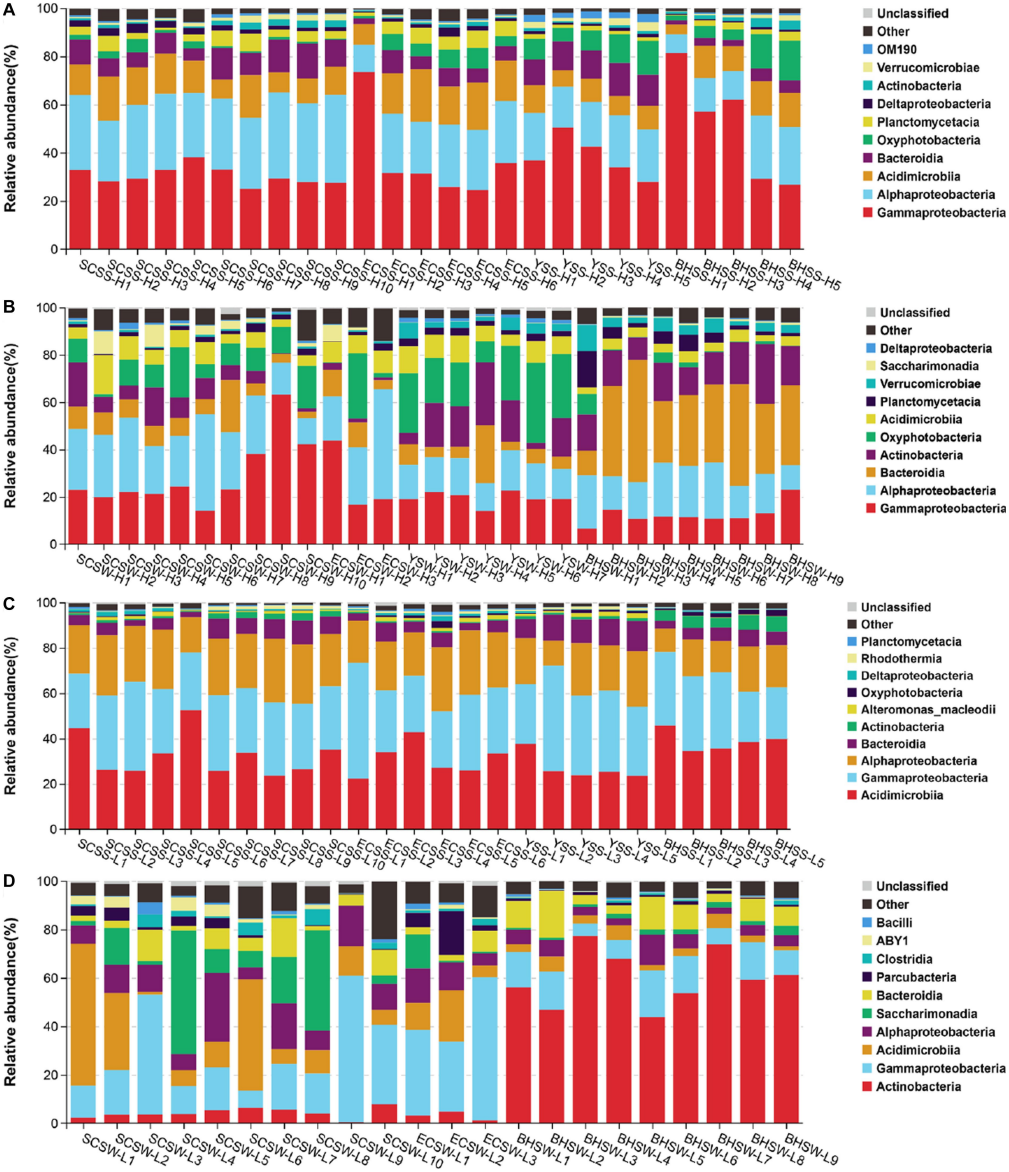
The composition of HNA and LNA bacterial communities at the class level, with the top ten class chosen in each sample. The operational taxonomic units (OTUs) were defined at 97% sequence similarity threshold. **(A)** The composition of HNA bacterial community in summer; **(B)** the composition of HNA bacterial community in winter; **(C)** the composition of LNA bacterial community in summer; **(D)** the composition of LNA bacterial community in winter. SCSS: samples from South China Sea in summer; SCSW: samples from South China Sea in winter; ECSS: samples from East China Sea in summer; ECSW: samples from East China Sea in winter; YSS: samples from Yellow Sea in summer; YSW: samples from Yellow Sea in winter; BHSS: samples from Bohai Sea in summer; BHSW: samples from Bohai Sea in winter.

A total of 6,551,727 sequences were obtained after quality control and rarefication, representing 11,714 OTUs for HNA bacteria and 8,879 OTUs for LNA bacteria, respectively. Overall taxonomic characterizations of HNA and LNA bacterial communities were conducted at the class level and the order level. At the class level, the HNA bacterial community was dominated by Alphaproteobacteria, Gammaproteobacteria and Acidimicrobiia, followed next by Bacteroidia in summer and dominated by Alphaproteobacteria, Gammaproteobacteria, Bacteroidia, and Acinobacteria in winter (Figures 2A,B). The LNA bacterial community was dominated by Acidimicrobiia, Gammaproteobacteria, Alphaproteobacteria, and Bacteroidia in summer, and dominated by Actinobacteria, Gammaproteobacteria, Acidimicrobiia and Alphaproteobacteria in winter (Figures 2C,D). At the order level, the HNA bacterial community was dominated by Rhodobacterales, Actinomarinales, Vibrionales, Alteromonadales and Flavobacteriales in summer (Supplementary Figure S6A), and dominated by Flavobacteriales, Rhodobacterales, Alteromonadales, Propionibacteriales and Actinomarinales in winter (Supplementary Figure S6B). The LNA bacterial community at the order level was dominated by Actinomarinales, SAR86_clade, SAR11_clade, Flavobacteriales, Rhodobacterales and Rhodospirillales in summer (Supplementary Figure S6C), and dominated by Frankiales, Actinomarinales, Betaproteobacteriales, Saccharimonadales, Flavobacteriales and Micrococcales in winter (Supplementary Figure S6D).

All OTUs of HNA and LNA bacteria were classified into three groups, namely, (i) exclusively HNA bacterial OTUs, (ii) exclusively LNA bacterial OTUs, and (iii) shared OTUs between HNA and LNA bacteria. The average numbers of OTUs of all samples classified as exclusive to HNA bacteria, exclusive to LNA bacteria, and shared between HNA and LNA bacteria in two seasons were 689, 369 and 574 OTUs, respectively. In each sampling site during the different seasons, the average number and fraction of exclusive and shared OTUs were different (Supplementary Table S3), indicating differences in the bacterial diversity of the three groups. A resolved phylogenetic classification at the order (top 20) levels also showed contrasting community compositions of the three groups over spatial and seasonal scales (Supplementary Figure S7). On the spatial scale, for example, in the exclusively LNA bacterial community, Isosphaerales, Leptospirales, Aeromonadales, Candidatus Peribacteria and Candidatus Komeilibact were the dominant groups in SCSS, Isosphaerales, Candidatus Azambacteria, Candidatus Falkowbacteria, Candidatus Peribacteria and Candidatus Pacebacteria were the dominant groups in ECSS, Isosphaerales, Aeromonadales and Candidatus Yanofskybacteria were the dominant groups in YSS, Saccharimonadales, Aeromonadales and Candidatus Peribacteria were the dominant groups in BHSS (Supplementary Figure S7C). Seasonally, in the exclusively LNA bacterial community, Saccharimonadales, Aeromonadales and Candidatus Peribacteria were the dominant groups in BHSS, whereas, Candidatus Kuenenbacteria, Candidatus Falkowbacteria, Leptospirales, Streptomycetales, Candidatus Magasanikbacteria, Candidatus Pacebacteria, Candidatus Yanofskybacteria were the dominant groups in BHSW (Supplementary Figure S7C). Although the shared OTUs were present in both LNA and HNA bacteria at the same site, there were some differences in the community composition between shared LNA and HNA bacteria. For example, Rhodobacterales, Flavobacteriales, Actinomarinales, Alteromonadales, Oceanospirillales, Propionibacteriales were the most abundant taxa in the shared HNA bacteria in SCSW (Supplementary Figure S7B), whereas Actinomarinales, Saccharimonadales and Pseudomonadales was more abundant in the shared LNA bacteria in SCSW (Supplementary Figure S7D).

**Figure 3:**
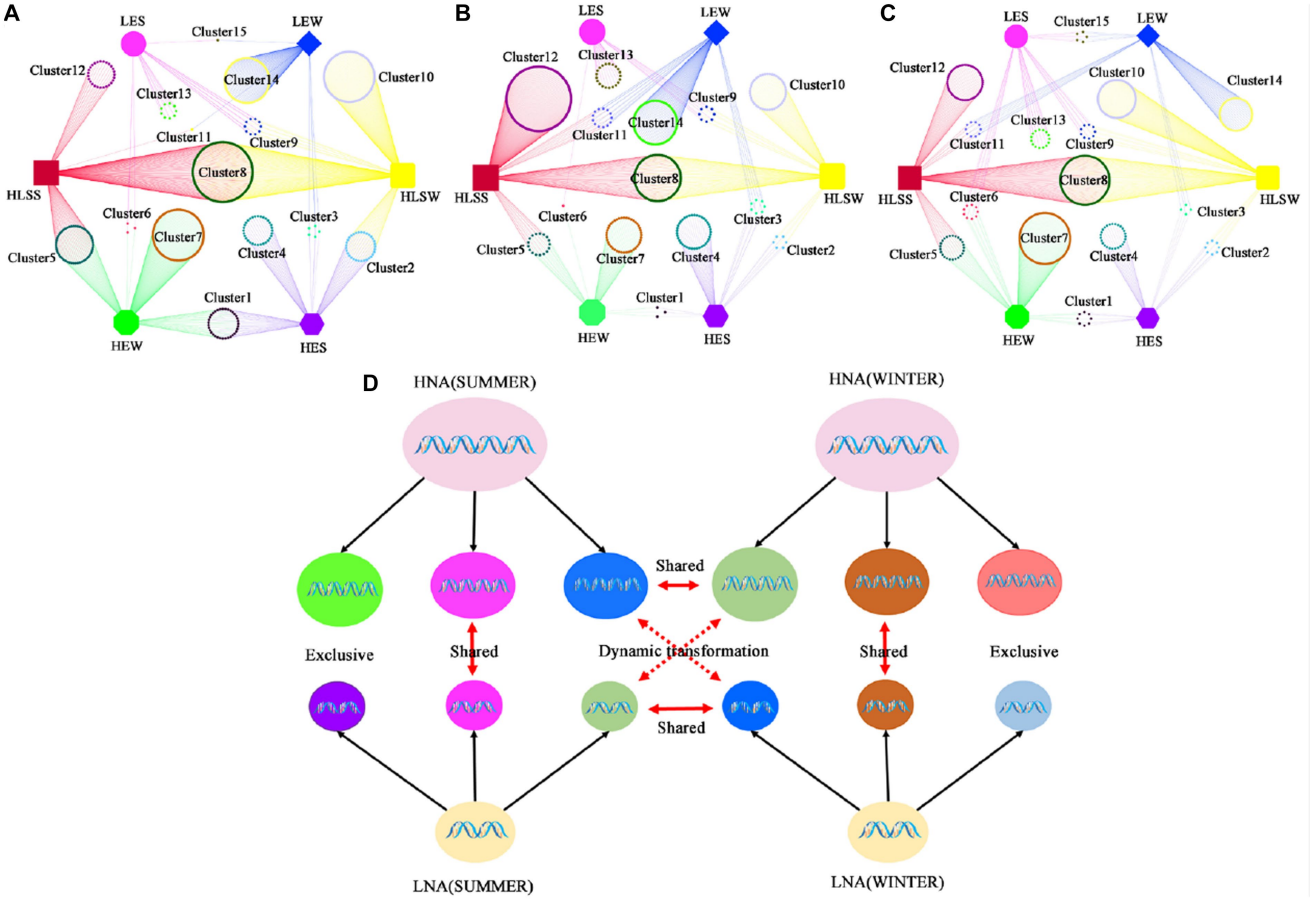
The transformations of LNA and HNA bacterial communities. **(A)** Samples from SCS in both seasons; **(B)** samples from ECS in both seasons; **(C)** samples from BHS in both seasons; **(D)** the model of the dynamic’s transformation of LNA and HNA bacterial communities. HES: exclusive HNA bacteria in summer; LES: exclusive LNA bacteria in summer; HLSS: shared between HNA and LNA bacteria in summer; HLSW: shared between HNA and LNA bacteria in winter; HEW: exclusive HNA bacteria in winter; LEW: exclusive LNA bacteria in winter.

Then, we analyzed the changes in the three groups at the same site in different seasons at the genus level (Figures 3A–C). Each group contains three fractions and each cluster was defined in Supplementary Table S4. Fraction 1 including cluster1 (exclusive HNA bacteria), cluster8 (shared between HNA and LNA bacteria) and cluster15 (exclusive LNA bacteria) appear in both seasons; Fraction 2 including cluster4 (exclusive HNA bacteria in summer), cluster7 (exclusive HNA bacteria in winter), cluster10 (shared between HNA and LNA bacteria in winter), cluster12 (shared between HNA and LNA bacteria in summer), cluster13 (exclusive LNA bacteria in summer), cluster14 (exclusive LNA bacteria in winter) was endemic to a particular group; Fraction 3 including cluster3 (shared between exclusive HNA bacteria in summer and exclusive LNA bacteria in winter) and cluster6 (shared between exclusive HNA bacteria in winter and exclusive LNA bacteria in summer) can dynamic exchange between LNA and HNA bacteria in accordance with changes in external conditions. Our data therefore suggest that LNA and HNA bacterial groups consist of three parts, the exclusive bacterial fractions, the shared bacterial fractions and the dynamic exchange fractions (Figure 3D).

### Alpha and beta diversity of LNA bacteria

**Figure 4:**
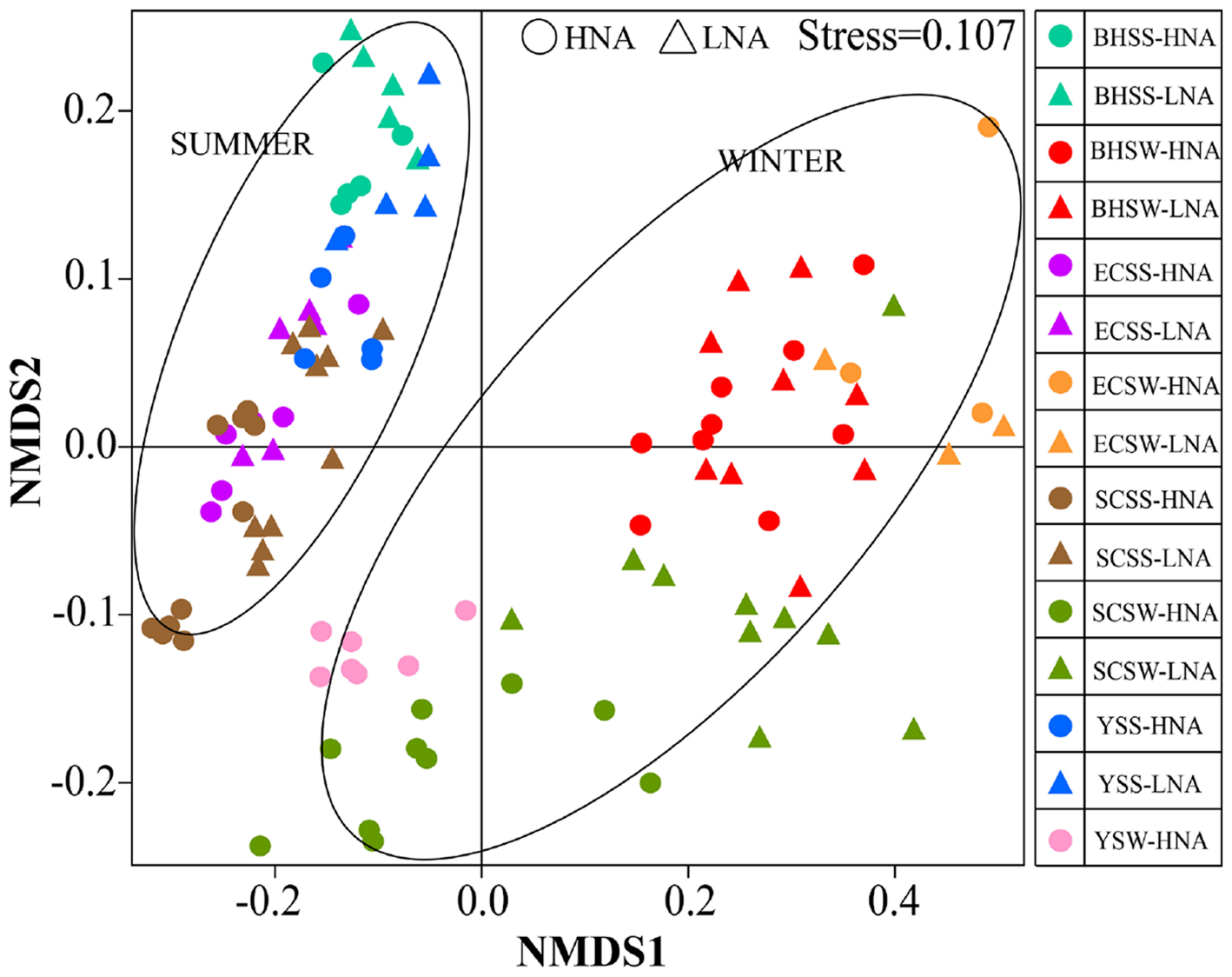
Non-metric multidimensional scaling (NMDS) of bacteria captured on two types of membrane filters calculated with weighted Unifrac dissimilarity from different site samples at different seasons. HNA bacteria represent the bacterial group captured on 0.45 μm membrane filters and LNA bacteria represent the bacterial group in the 0.45 μm-filtrate that is subsequently captured on 0.1 μm membrane filters. SCSS: samples from South China Sea in summer; SCSW: samples from South China Sea in winter; ECSS: samples from East China Sea in summer; ECSW: samples from East China Sea in winter; YSS: samples from Yellow Sea in summer; YSW: samples from Yellow Sea in winter; BHSS: samples from Bohai Sea in summer; BHSW: samples from Bohai Sea in winter.

In the present study, the bacterial diversity indices (Shannon and Simpson) for LNA bacteria were relatively lower than those for HNA bacteria in both seasons (Supplementary Figure S8), indicating that LNA bacteria was less diverse than HNA bacteria in terms of bacterial diversity. To explore the overall variability in community composition, we performed non-metric multidimensional scaling (NMDS) analysis, which revealed the compositional dissimilarities between HNA and LNA bacterial communities in different sites and seasons. A significant season-to-season and site-to-site separations was observed in HNA and LNA bacteria, respectively (Figure 4), which was supported by the PERMANOVA analysis (Supplementary Table S6). And the significant cluster-to-cluster variations between HNA and LNA bacteria in different sites were also observed except in the Bohai sea samples in winter (Figure 4). Overall, the spatiotemporal changes had significant effects on HNA and LNA bacterial community structure.

### Factors shaping HNA and LNA bacterial communities

**Figure 5:**
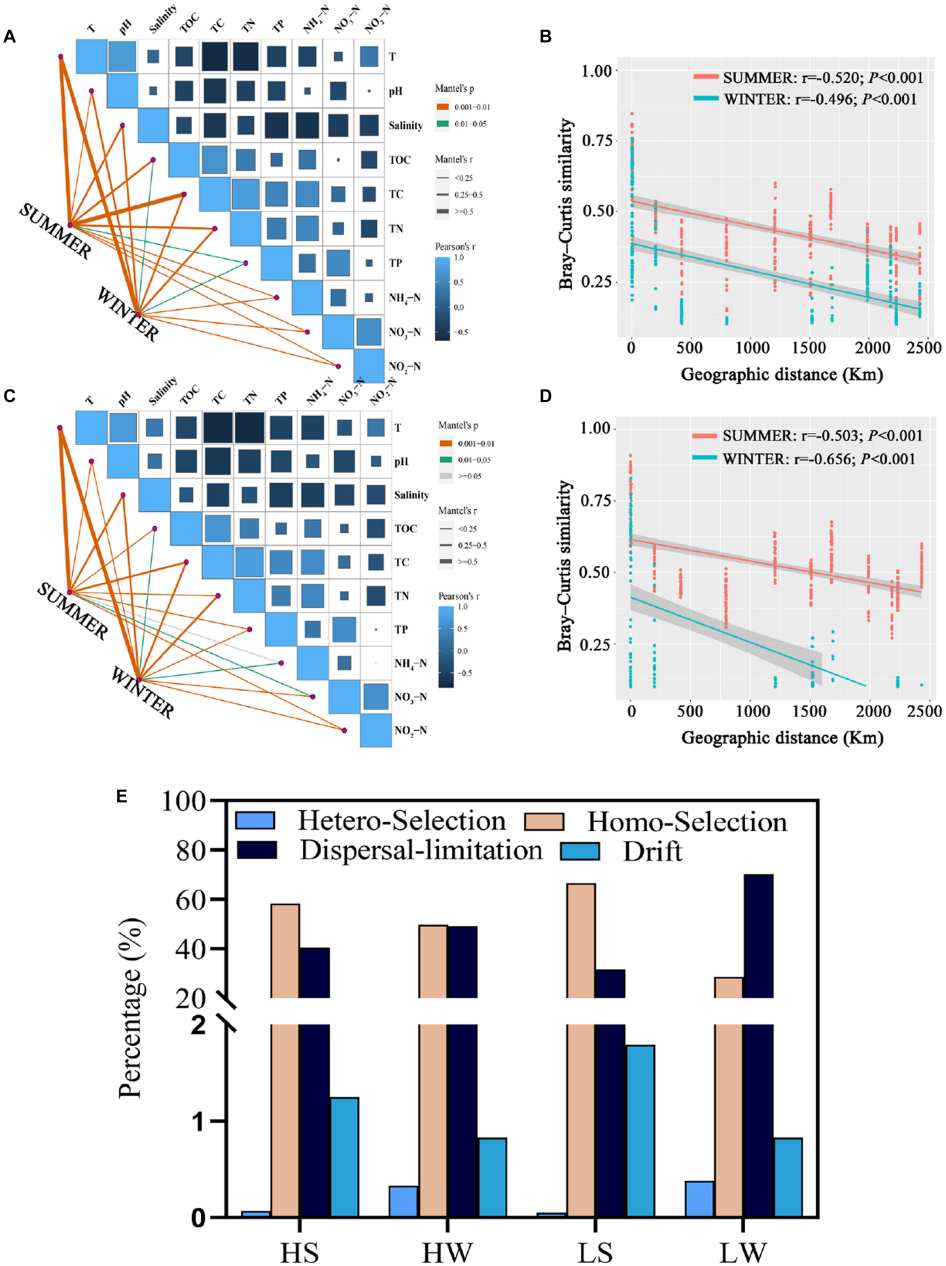
Drivers of variation in HNA and LNA bacteria. **(A,C)** Mantel analysis examining the relationships between environmental physicochemical properties and the composition of HNA **(A)** and LNA **(C)** bacterial communities in both seasons. **(B,D)** Distance–decay patterns of HNA **(B)** and LNA **(D)** bacterial communities in both seasons. **(E)** Null model analysis revealing the relative contribution of different ecological processes on HNA and LNA bacterial communities in both seasons. HS: HNA bacteria in summer; HW: HNA bacteria in winter; LS: LNA bacteria in summer; LW: LNA bacteria in winter.

In order to identify environmental drivers in HNA and LNA bacterial communities, we correlated the communities of HNA and LNA bacteria in two seasons with the environmental factors (Figures 5A,C). It is found that all environmental factors were significantly associated with HNA and LNA bacterial communities in two seasons (*p* < 0.05, Figures 5A,C) except that between NH_4_-N and LNA bacteria in summer (*p* > 0.05, Figure 5C). Additionally, the observation of bacterial assemblages was reflected by a clear distance-decay pattern. The Spearman’s correlation between the Bray–Curtis similarity and geographic distance showed significantly negative correlations for the bacterial community with a correlation coefficient of −0.520, −0.496, −0.503, and − 0.656 (*p* < 0.01) for HNA bacteria in summer, HNA bacteria in winter, LNA bacteria in summer and LNA bacteria in winter (Figures 5B,D). These results indicated that the similarity in HNA and LNA bacterial community composition between any two regions or sites decreased with increasing geographical distances.

To explore mechanisms underpinning the observed geographic pattern, neutral processes in community assembly were analyzed. The results showed that homogeneous selection and dispersal limitation were the most important process. For example, homogeneous selection accounted for 58.17, 49.65, 66.61 and 28.67% of the community variation of HNA bacteria in summer, HNA bacteria in winter, LNA bacteria in summer and LNA bacteria in winter. Dispersal limitation accounted for 40.51, 49.19, 31.55 and 70.12% of the community variation of HNA bacteria in summer, HNA bacteria in winter, LNA bacteria in summer and LNA bacteria in winter, respectively (Figure 5E). Homogeneous selection was more important in structuring HNA and LNA bacterial communities than heterogeneous selection in both seasons (Figure 5E), which suggests that the divergence of HNA and LNA bacterial community compositions is constrained. Additionally, both homogeneous selection and dispersal limitation showed seasonal dependent effect on the LNA and HNA microbial communities (Figure 5E).

The Mantel and partial Mantel analysis showed that LNA bacterial community was primarily governed by spatial factors in both seasons (Supplementary Figures S9C,D), which means that stochastic (spatial) factors exert a greater effect on LNA bacterial community than deterministic (environmental) factors. And deterministic (environmental) factors explained more variation in HNA bacterial distribution than stochastic (spatial) factors in summer (Supplementary Figure S9A) and stochastic (spatial) factors exert a greater effect in HNA bacterial distribution than deterministic (environmental) factors in winter (Supplementary Figure S9B).

### The co-occurrence networks of HNA and LNA bacteria

**Figure 6:**
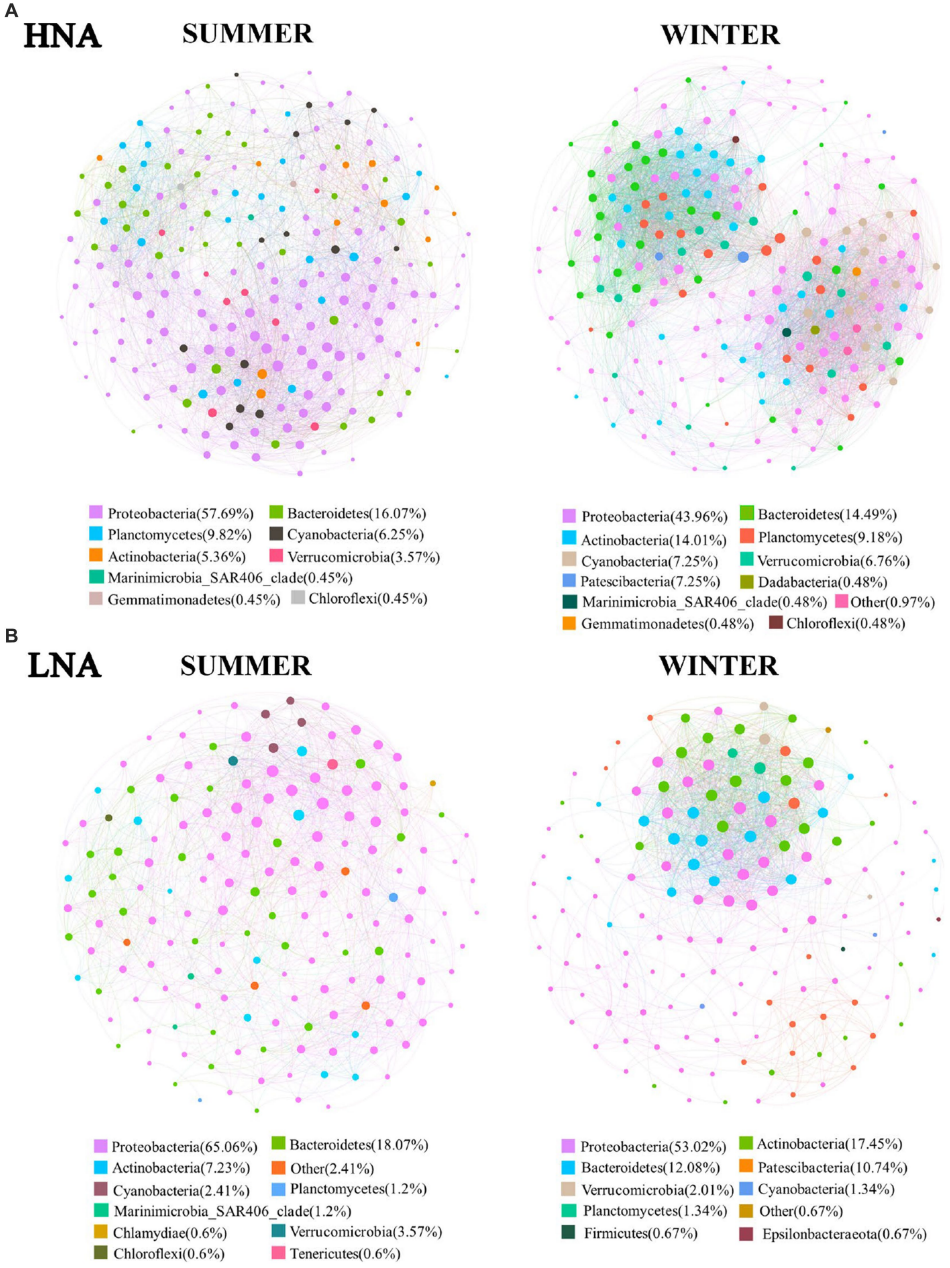
Co-occurrence networks of HNA and LNA bacterial community based on pairwise Spearman’s correlations between OTUs. **(A)** The network of HNA bacteria; **(B)** the network of LNA bacteria. Each shown connection has a correlation coefficient > |0.6| and a *p* value <0.01.

In the present work, we explored the co-occurrence patterns across the communities of HNA and LNA bacteria in network analysis, respectively (Figure 6). And the nodes number of the co-occurrence network across the communities of HNA and LNA bacteria were shown in Supplementary Table S7. For HNA bacteria, most of the nodes in the networks belonged to the phyla of Proteobacteria, Bacteroidetes, and Actinobacteria in summer, and Proteobacteria, Bacteroidetes, Actinobacteria, Planctomycetes and Cyanobacteria in winter (Figure 6A). Whereas, most of the nodes belonged to the phyla of Proteobacteria, Bacteroidetes, Planctomycetes, and Cyanobacteria in the network of LNA bacterial in summer and Proteobacteria, Actinobacteria, Bacteroidetes, and Patescibacteria in winter (Figure 6B). And then, the percentage of each subclass of Proteobacteria in the co-occurrence networks was analyzed. The results showed that a higher percentage of Gammaproteobacteria was found in all the co-occurrence networks than Alphaproteobacteria and Deltaproteobacteria (Supplementary Figure S10). Those results indicated that Gammaproteobacteria occupy an important position in the network and play a key role in mediating network interactions and maintaining the overall metabolic function of HNA and LNA bacterial communities in marine environment.

The complexity of connections in the co-occurrence networks of HNA and LNA bacteria in the two seasons were also explored, and the parameters that represent the network topological structure were calculated (Supplementary Table S7). The topological features of the network revealed that the network connectivity of HNA bacterial community (links: 5,244) in winter were higher than that in summer (links: 3,645), which indicated that the interactions in HNA bacteria community were more complex in winter than that in summer. Whereas the network connectivity of LNA bacterial community (links: 1,880) in summer were higher than that in winter (links: 1,466), which indicated that the interactions in LNA bacteria community were more complex in summer than that in winter. The weighted average degree of the co-occurrence networks of HNA than LNA bacteria in winter were higher than that in summer (Supplementary Table S7), indicating that the co-occurrence networks of HNA and LNA bacteria in winter were more complex than that in summer. Additionally, higher weighted average degree was found in the co-occurrence networks of HNA than that of LNA bacteria in the same season (Supplementary Table S7), indicating that the co-occurrence networks of HNA bacteria was more complex than those of LNA bacteria in the same season.

### The ecological functions of LNA and HNA bacteria

**Figure 7:**
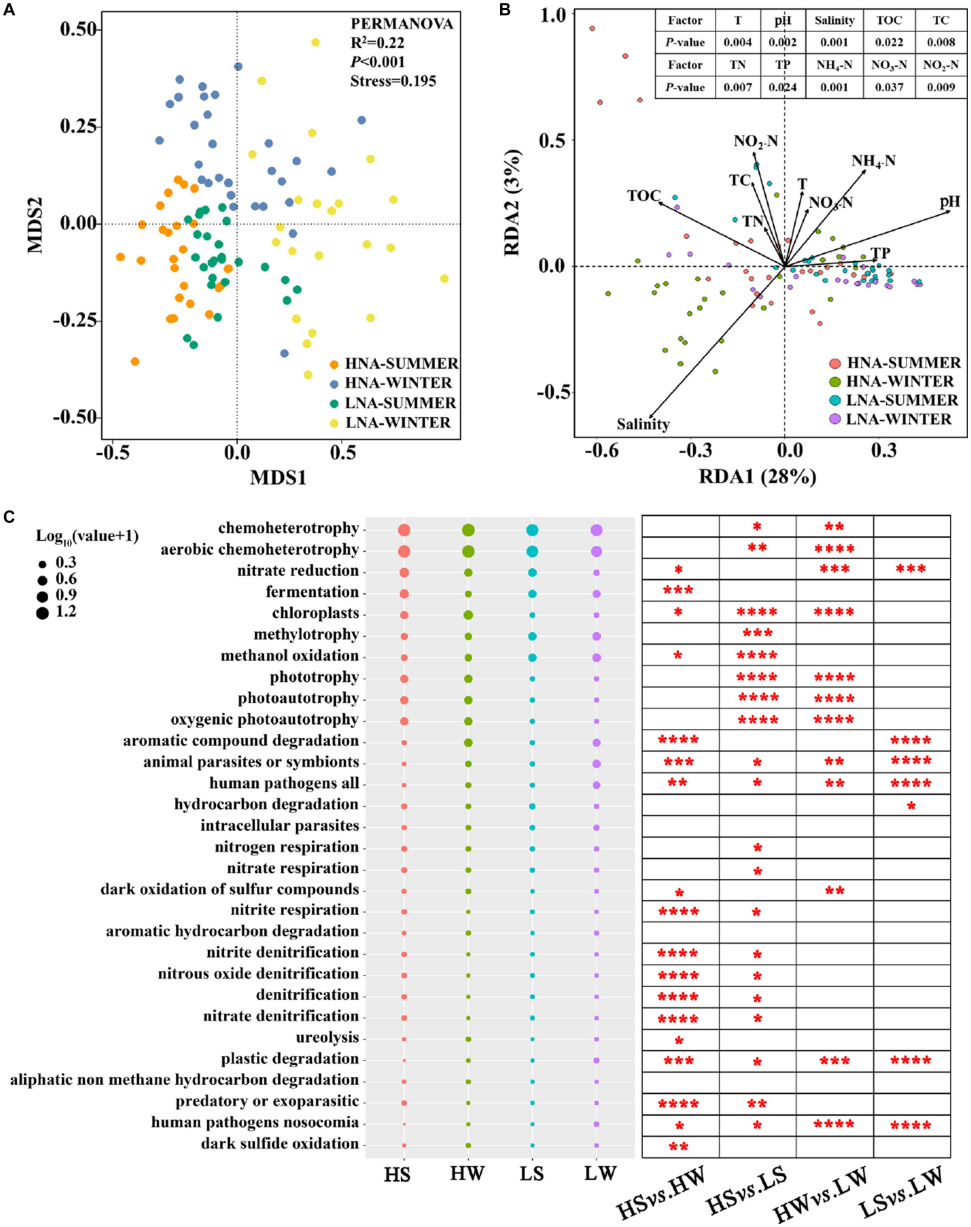
The functions of HNA and LNA bacterial communities in summer and winter. **(A)** Non-metric multidimensional scaling (NMDS) analysis showing that the significantly distinct of HNA and LNA bacteria were detected by PERMANOVA. **(B)** Redundancy analysis (RDA) between HNA and LNA bacterial functions and environmental factors. **(C)** The relative abundance of the top 30 bacterial functions analyzed by FAPROTAX and comparison of HNA and LNA bacterial functions in two seasons. The superscripts (^*^, ^**^, ^***^, and ^****^) represent significant (*p* < 0.05, *p* < 0.01, *p* < 0.001, and *p* < 0.0001) dissimilarity between the two group. HS: HNA bacteria in summer; HW: HNA bacteria in winter; LS: LNA bacteria in summer; LW: LNA bacteria in winter.

The functions of LNA and HNA bacteria were predicted by FAPROTAX analysis (Li et al., 2021). Non-metric multidimensional scaling (NMDS) analysis revealed that significant differences existed among the functional compositions of HNA bacteria in summer, HNA bacteria in winter, LNA bacteria in summer and LNA bacteria winter (Figure 7A) and PERMANOVA further verified the significant differences among them (*p* < 0.001; Figure 7A). Redundancy analysis (RDA) was used to perform ordination to analyze the relationships between the functions of the four groups with environmental variables. The results showed that the environmental factors, such as: T (*p* < 0.01), pH (*p* < 0.01), salinity (*p* < 0.01), TOC (*p* < 0.05), TC (*p* < 0.01), TN (*p* < 0.01), TP (*p* < 0.05), NH_4_-N (*p* < 0.01), NO_3_-N (*p* < 0.05), NO_2_-N (*p* < 0.01), had a significant effect on the functions of the four groups (Figure 7B). Comparing the relative abundance of the ecological functions showed that several functions, including chemoheterotrophy, aerobic chemoheterotrophy, chloroplasts, phototrophy, photoautotrophy, oxygenic photoautotrophy, animal parasites or symbionts, pathogenicity (including all human pathogens, nosocomial human pathogens) and plastic degradation, were differed significantly between HNA and LNA bacteria (Figure 7C). Seasonal changes also have a significant impact on the ecological functions of HNA and LNA bacteria, including functions such as nitrate reduction, aromatic compound degradation, animal parasites or symbionts, all human pathogens, plastic degradation, and nosocomial human pathogens (Figure 7C).

## Discussion

### Environmental factors significantly influenced the cell size, cell count, percentage and distributions of HNA and LNA bacteria

Previous studies have reported that HNA bacteria tend to grow in eutrophic and mesotrophic environments, while LNA bacteria tend to grow in oligotrophic environments (Jochem et al., 2004; Andrade et al., 2007). In the present study, we found that the cell count and percentage of HNA and LNA bacteria were significantly correlated with different environmental variables (Supplementary Figure S4). The similar phenomenon has been reported in the previous studies (Liu et al., 2016, 2017). In the present study, the influence of T and salinity on the count of HNA and LNA bacteria were stronger than that of nutrient conditions (Supplementary Figure S4). This result is consistent with the previous research that seasonal changes in environmental variables have a more significant effect on microbial community patterns than nutrients (Gilbert et al., 2012; Read et al., 2015). And the percentage of HNA and LNA bacteria showed significantly positive and negative correlation with T, respectively (Supplementary Figure S4), which indicated that in terms of growth potential, LNA bacteria were less competitive than HNA bacteria in coping with temperature changes (Song et al., 2019a). In addition, we found a strong positive correlation between the TC and TN with the percentage and FSC median values of LNA bacteria (Supplementary Figures S4, S5), which indicated that the availability of nutrients is an important factor affecting and restricting bacterial growth (Egli, 2010). High concentration of nitrogen can effectively promote the growth and reproduction of bacterial communities in the environments (Song et al., 2019b). As a necessary nutrient for microbial growth, carbon source also has an important impact on bacterial growth (Egli, 2010). In the natural environment, most carbon sources are in the polymer state and cannot be effectively absorbed by bacteria (Hedges, 1992). Only a small part of dissolved organic carbon sources is utilized by bacteria. In the oligotrophic state, available organic carbon sources are even lower (Egli, 1995). Therefore, the increase of carbon source concentration can effectively promote the growth and metabolic activity of bacteria. Previous studies have suggested that the HNA:LNA ratio provides an indirect indication that most of the populations are considered to be active (Sharuddin et al., 2018). In the present study, the HNA:LNA ratio was significantly negatively correlated with NH_4_-N, TN and TC (*p* < 0.05), respectively (Supplementary Figure S4). The results indicated that LNA bacteria are sensitive to the changes of nutrients, and the increase of nutrients can promote the growth of LNA bacteria. Which also suggests that they may play different ecological roles in the same ecosystem (Liu et al., 2016).

Additionally, we found that the spatial and temporal variation, as well as the interactions between those two factors also affects the cell size, cell count and percentage of LNA and HNA bacteria (Supplementary Table S2), which is consistent with previous studies (Nishimura et al., 2005; Liu et al., 2016, 2017; Eswaran and Khandeparker, 2021). This may be due to the seasonal pattern of activity of HNA and LNA bacteria, which leads to the changes in cell size and percentage of HNA and LNA bacteria (Worden et al., 2004).

### Different diversity but shifting between HNA and LNA bacteria

Bacterial populations that can be distinguished by the cell size and nucleic acid content have been found consistently using flow cytometry, and have raised the question of whether they have the same phylogeny. In the present study, we observed that the diversity differed substantially between HNA and LNA bacteria. Previous study reported that Rhodobacterales, SAR116 and Bacteroidetes contributed primarily to the HNA bacteria, whereas other groups such as SAR11 and SAR86 contributed largely to the LNA bacteria (Vila-Costa et al., 2012). Here, however, our results showed that bacterial taxa at the order level differ between HNA and LNA bacteria (Supplementary Figure S6), which was supported by the PERMANOVA analysis (Supplementary Table S6), although they have similar composition and dominated by Alphaproteobacteria, Gammaproteobacteria Acidimicrobiia and Bacteroidia at the class level (Figure 2). In the present study, we found some major tax contributed to only one group (Supplementary Figure S6), such as: SAR11 only contributed primarily to LNA bacteria in summer. Previous studies had been observed that the SAR11 contributed to the LNA bacteria at oceanic sites (Longnecker et al., 2005; Schattenhofer et al., 2011). This group is abundant and ubiquitous in the surface waters of the ocean (Morris et al., 2002) and contains small and efficient genomes (Giovannoni et al., 2005). In addition, the Gammaproteobacterial SAR86 group, particularly in summer, contributed primarily to HNA and LNA bacteria. This phenomenon was observed in the previous studies. For example, SAR86 cells was present in LNA bacteria in the Celtic Sea and the northern North Sea (Zubkov et al., 2001, 2002) and in HNA bacteria in North Atlantic waters (Schattenhofer et al., 2011).

In this study, we found that a seasonal difference exists in HNA and LNA bacterial communities. For example, the summer LNA bacterial community was dominated by Actinomarinales, SAR86, SAR11, Flavobacteriales, Rhodobacterales and Rhodospirillales (Supplementary Figure S6C), while the winter LNA bacterial community was mainly composed by Frankiales, Actinomarinales, Betaproteobacteriales, Saccharimonadales, Flavobacteriales and Micrococcales (Supplementary Figure S6D), a pattern that had been observed in the exclusively LNA bacterial community (Supplementary Figure S7C). This phenomenon was observed in the previous study that HNA bacterial community was dominated by Rhodobacterales in summer while dominated by Bacteroidetes in winter (Vila-Costa et al., 2012). In addition, seasonal differences have also been found in other ecosystems, such as bacterial community in freshwater (Paruch et al., 2020), archaeal community in coastal sediments (Liu et al., 2020). Overall, these results indicate a strong phylogenetic signal in the composition of HNA and LNA bacteria (Vila-Costa et al., 2012).

The four alternative hypotheses were raised to explain the cytometric signatures of natural communities by Bouvier and colleagues in 2007. They proposed that: first, HNA bacteria are the active and growing cells, and LNA bacteria are composed of the inactive, dormant, injured or dead cells of HNA bacteria. Second, HNA bacteria are composed of the active LNA bacteria that are undergoing rapid cell division and have copied DNA or acquired multiple copies of the genome. Third, HNA and LNA bacteria are completely different groups with independent intrinsic characteristics and little or no interaction with each other. Fourthly, HNA and LNA bacteria have their own unique bacterial groups, but there are dynamic transitions between them. Our data better support the third and fourth scenarios proposed by Bouvier et al. (2007) whereby the clusters such as those in fraction 2 have fidelity to a specific group, whereas some clusters such as those in fraction 3 are equally likely to appear in HNA and LNA bacteria, leading to a switch of bacterial cells from one group to the other (Bouvier et al., 2007). Similar results have been reported by Vila-Costa and colleagues (Vila-Costa et al., 2012). Several biological mechanisms were reported that could explain the passage of cells from one group to the other, such as DNA replication and reduction during cell division (Lebaron and Joux, 1994), increase in DNA content during high activity periods (Gasol et al., 1999), DNA degradation during starvation (Joux et al., 1996), cellular inactivation by grazers or other factors (Gasol et al., 1999), varying degrees of polyploidy (Binder and Chisholm, 1995), or adaptive changes in genome size over extremely short time-scales (Nilsson et al., 2005). In addition, we also found that some taxa in the community composition of these three groups exhibited seasonal preference. For example, cluster12 (shared between HNA and LNA bacteria), cluster13 (exclusive LNA bacteria) and cluster4 (exclusive HNA bacteria) were found in summer. In contrast, cluster10 (shared between HNA and LNA bacteria), cluster14 (exclusive LNA bacteria) and cluster7 (exclusive HNA bacteria) were found in winter (Figures 3A–C). This phenomenon has been reported in a previous study and was explained by the seasonal differentiation for some bacterial groups being subjected to a strong seasonality in a coastal marine environment (Auladell et al., 2021).

LNA bacteria was less diverse than HNA bacteria in terms of bacterial diversity in this study (Supplementary Figure S8), which was in line with the previous studies (Vila-Costa et al., 2012; Song et al., 2019b). In the present study, the significant difference was found between HNA and LNA bacteria by the PERMANOVA analysis (Figure 4; Supplementary Table S6), indicating that LNA and HNA bacteria separated by 0.45 μm filtration revealed dramatic differences in the community. The results were observed in the previous study (Song et al., 2019b). While there was a clear separation between the two groups, HNA and LNA bacteria were not completely independent, which may be due to the shared OTU between them (Proctor et al., 2018). This phenomenon was also observed in the previous study that the separation through cell sorting failed to see a clear separation of LNA and HNA bacterial communities (Longnecker et al., 2005). Additionally, spatiotemporal changes also had significant effects on HNA and LNA bacterial community structure (Figure 4; Supplementary Tables S5, S6), which indicated that HNA and LNA taxonomy is dependent on location and time (Proctor et al., 2018).

### Mechanisms controlling the assembly of HNA and LNA bacterial communities

The differences in community composition of HNA and LNA bacteria attributed to the cell size and other factors. To investigate the formation mechanism of HNA and LNA bacterial communities, we investigated the environmental factors that may drive the variation of the bacterial communities. Environmental factors (such as: T, salinity, TOC, TC, TN, TP) were significantly related to variations in HNA and LNA bacteria in both season (Figures 5A,C). Those results revealed that environmental factors played key roles in shaping HNA and LNA bacterial communities, which is consistent with a previous study (Song et al., 2019b). Previous spatiotemporal analysis has shown that nutrient concentrations strongly affected bacterial composition because they play crucial roles for the growth and development of bacteria (Wang et al., 2015). However, in the present study, temperature played a more important role in determining HNA and LNA bacterial communities than nutrients (TOC, TC, TN and TP) or salinity in the two seasons (Figures 5A,C). This is consistent with a previous finding that temperature is the major environmental factor shaping microbial community composition in marine environment (Sunagawa et al., 2015). The distance-decay pattern has frequently been found to underly marine microbial spatial dynamics (Zinger et al., 2014). Here, significant negative relationships between the geographical distance and Bray–curtis similarity of the HNA bacterial community as well as the LNA bacterial community were observed in both seasons (*p* < 0.001; Figures 5B,D). Together, our data support biogeographic patterns of microbial communities, in line with previous studies (Wu et al., 2018; Chen et al., 2019; Liu et al., 2020). Previous studies reported that the significant distance-decay patterns are associated with many ecological processes. For example, species sorting that is adaptation to local environments can also lead to distance-decay in community similarity (Hanson et al., 2012) and any dispersal limitation of microeukaryotes should lead to a decrease in community similarity (Zhang et al., 2018). Next, we explored the relative importance of deterministic and stochastic processes in structuring HNA and LNA bacterial communities. Previous studies reported that the relative importance of stochastic and deterministic assembly processes in shaping microbial communities also varied through space and time (Stegen et al., 2013; Zhou, 2017), which was also observed in the present study (Supplementary Figure S9). In addition, stochastic (spatial) factors exert a greater effect on the LNA bacterial community than deterministic (environmental) factors in summer (Supplementary Figure S9C), whereas deterministic (environmental) factors explained more variation in HNA bacterial distribution than stochastic (spatial) factors in summer (Supplementary Figure S9A). Similar results were found that deterministic processes governed community turnover in soil bacteria while the community turnover of soil fungi from the same samples was mostly influenced by stochastic processes (Powell et al., 2015). Those results indicated that deterministic and stochastic processes paly different roles in the different microbial groups.

Previous studies have reported that the combination of ecological selection, organismal dispersal, ecological drift, and speciation has an important role in ecological community construction (Logares et al., 2018; Vass et al., 2019). Selection has been confirmed to be the prevalent process structuring bacterial communities across various ecosystems (Lindström and Langenheder, 2011; Hanson et al., 2012). Then we explored the role of different ecological processes in HNA and LNA bacterial community construction (Figure 5E). In the present study, homogeneous selection accounted for a high proportion of HNA and LNA bacterial community variation in summer (Figure 5E). The proportion of homogeneous selection in the present study was higher than that reported for prokaryotes in surface-ocean (Logares et al., 2020), which may be explained by the phenomenon that several consistent environmental factors selected for similar microbial communities across samples (Liu et al., 2020). Homogeneous selection was more important in structuring HNA and LNA bacterial communities than heterogeneous selection in both seasons (Figure 5E), which suggests that the divergence of HNA and LNA bacterial community compositions is constrained (Zhou, 2017). Even though the SCS, ECS, YS and BHS are connected, they have separate surface currents that may serve as a barrier and limit dispersal (Wu et al., 2017). Thus, we found that the proportion of dispersal limitation in the present study was higher than that reported for marine bacteria in the East China sea (Wu et al., 2018). Additionally, both homogeneous selection and dispersal limitation showed seasonal dependent effect on the LNA and HNA microbial communities (Figure 5E). These results indicate that seasonal changes also affect the contribution of ecological processes to bacterial community construction (Stegen et al., 2012).

### The co-occurrence networks and ecological functions of HNA and LNA bacteria

In theory, the relationship between species is primarily driven by three ecological processes: environmental filtration, diffusion restriction, and interactions between organisms (Li et al., 2021; Yuan et al., 2021). The interaction between organisms is the main driving force of network topology. In ecosystems, the interactions between microbes are formed by the exchanges of materials, energy and information (Montoya et al., 2006), which can be affected by abiotic and biotic factors, including predation, competition, mutualism, commensalism, and parasitism (Faust and Raes, 2012). Co-occurrence patterns, as a method of revealing complex microbial community relationships, have been widely used in the interspecific analysis of microbial communities in marine and other environments (Li et al., 2021; Yuan et al., 2021; Zhang et al., 2021). In the present study, most of the nodes in the networks of HNA and LNA bacteria in both seasons belonged to the phyla of Proteobacteria (Figure 6), which is consistent with a previous study (Zhang et al., 2021). It is worth noting that a higher percentage of Gammaproteobacteria was found in all the co-occurrence networks than Alphaproteobacteria and Deltaproteobacteria (Figure S10), indicating that Gammaproteobacteria play key roles in the co-occurrence networks. As previous studies reported that Gammaproteobacteria, such as *Pseudomonas, Alteromonas, Serratia, and Methylobacter*, are ubiquitous worldwide and have been previously identified as a dominant group in the marine environment (Orcutt et al., 2011; Li et al., 2014) and have an important ecological role in the detrital carbon cycle (Fu et al., 2020; Hiraoka et al., 2020). In the present study, more than half of the links in the co-occurrence network of HNA and LNA bacteria across the two seasons were positively correlated (Supplementary Table S7). Previous studies reported that positive correlations between nodes indicate the existence of cooperative behaviors such as cross-feeding, syntrophic interactions, mutualistic interactions and commensalism as well as shared environmental requirements and common dispersal barriers (Fuhrman, 2009; Berry and Widder, 2014). Whereas, negative correlations between nodes may originate from a wide range of co-exclusion mechanisms, including competition for limited resources and distinctive environmental niches (Fuhrman, 2009; Berry and Widder, 2014). The results in this study means that the relationships between them tended towards co-occurrence rather than co-exclusion (Berry and Widder, 2014).

Microbial metabolism fuels the most elemental biogeochemical cycles on the earth. It was reported that environmental conditions strongly affect the distribution of functional groups in marine microbial communities through the formation of metabolic niches (Louca et al., 2016), thus affecting the metabolic and ecological functions of microorganisms. In the present study, the significant differences existed among the functional compositions of HNA and LNA bacteria in both seasons (Figures 7A,C), which indicated that LNA and HNA bacteria had distinct ecological functions (Song et al., 2019b). The reason is that HNA and LNA bacteria are mainly attributed to different dominant and unique bacterial groups, thus giving rise to different ecological functions (Song et al., 2019b). For example, previous studies have revealed that Alphaproteobacteria and Gammaproteobacteria had a higher relative contribution to the ecological functions including photoautotrophy, aerobic chemoheterotrophy and anoxygenic photoautotrophy, whereas Actinobacteria and Acidimicrobiia had a lower relative contribution to them (Louca et al., 2016). These results are in line with the present study that Alphaproteobacteria and Gammaproteobacteria were the dominant class within HNA bacteria, Actinobacteria and Acidimicrobiia were the dominant class within LNA bacteria. In addition, seasonal changes also have a significant impact on the ecological functions of HNA and LNA bacteria (Figure 7C). Previous study also reported that seasonal changes showed the greatest impact on the functional genes group (Zhang et al., 2019). This may be due to the reason that environmental conditions strongly influence the distribution of functional groups in marine microbial communities by shaping metabolic niches (Louca et al., 2016). For example, previous studies had revealed that Actinobacteria had a higher relative contribution to the ecological functions, including nitrate reduction, nitrate respiration than Acidimicrobiia (Louca et al., 2016), which is in line with the present study that Acidimicrobiia was the dominant class in summer and Actinobacteria was the dominant class in winter within LNA bacteria.

## Conclusion

This study reveals the community composition, geographic distribution, underlying mechanism, co-occurrence relationship and ecosystem functions of HNA and LNA bacteria using samples collected from Chinese coastal seawaters. The results showed that the HNA bacterial community was dominated by Rhodobacterales, Actinomarinales, Vibrionales in summer and by Flavobacteriales, Rhodobacterales, Alteromonadales in winter. The LNA bacterial community was dominated by Actinomarinales, SAR86, SAR11 in summer and by Frankiales, Actinomarinales, Betaproteobacteriales in winter. Some bacterial taxa are predictably present in a specific group while other taxa are equally likely to appear in HNA and LNA fractions, which implies a switch of bacterial cells from one group to the other. The bacterial diversity indices for LNA bacteria were relatively lower than those for HNA bacteria in both seasons. The communities of HNA and LNA bacteria exhibited clear spatiotemporal patterns and significant distance-decay patterns that were consistent with season. Furthermore, a quantitative assessment of ecological processes revealed that dispersal limitation and homogeneous selection exerted important roles in the community assembly of HNA and LNA bacteria. Additionally, the co-occurrence relationships were different between HNA and LNA bacteria and changed over seasons. Furthermore, the ecological functions, such as chemoheterotrophy, phototrophy, photoautotrophy, oxygenic photoautotrophy, were significantly different between HNA and LNA bacteria. Our study has laid a foundation for understanding the spatiotemporal dynamics of HNA and LNA bacteria in Chinese coastal seawaters, as well as for reinforcing ideas about a wide range of ecological and evolutionary functions of HNA and LNA bacteria. Furture studies will focus on exploring the functional properties of HNA and LNA bacteria based on multi-omics techniques, as well as discovering more functional microorganisms.

## Data availability statement

The datasets presented in this study can be found in online repositories. The names of the repository/repositories and accession number(s) can be found in the article/Supplementary material.

## Author contributions

WH: data curation and writing-original draft preparation. NZ: visualization. YZ: software. MB: writing-review and editing, and funding acquisition. YW: supervision, writing-review and editing, and funding acquisition. All authors contributed to the article and approved the submitted version.

## Funding

This study was supported by Shenzhen Natural Science Foundation (JCYJ20220530164606013), the 111 program, Ministry of Education, China (T2017002) and the Tip-top Scientific and Technical Innovative Youth Talents support plan.

## Conflict of interest

The authors declare that the research was conducted in the absence of any commercial or financial relationships that could be construed as a potential conflict of interest.

## Publisher’s note

All claims expressed in this article are solely those of the authors and do not necessarily represent those of their affiliated organizations, or those of the publisher, the editors and the reviewers. Any product that may be evaluated in this article, or claim that may be made by its manufacturer, is not guaranteed or endorsed by the publisher.

## Footnotes

^1^ https://gephi.org
