# Supplementary material for: Spatiotemporal dynamics of high and low nucleic acid-content bacterial communities in Chinese coastal seawater: assembly process, co-occurrence relationship and the ecological functions
Source: Front Microbiol. 2023 Aug 2;14:1219655. doi: 10.3389/fmicb.2023.1219655 (PMC10433394; doi:10.3389/fmicb.2023.1219655)
Supplement: Supplementary file 1 [file Data_Sheet_1.pdf]

Supplement material

Spatiotemporal dynamics of high and low nucleic acid-content bacterial communities in Chinese coastal seawater: assembly process, co-occurrence relationship and the ecological functions

Wei Hu[1], Ningning Zheng[1], Yadi Zhang[1], Mark Bartlam[2], Yingying Wang[1,*]

[1]Key Laboratory of Pollution Processes and Environmental Criteria (Ministry of Education), Tianjin Key Laboratory of Environmental Remediation and Pollution Control, College of Environmental Science and Engineering, Nankai International Advanced Research Institute (Shenzhen Futian), Nankai University, Tianjin, China

[2]State Key Laboratory of Medicinal Chemical Biology, College of Life Sciences, Nankai International Advanced Research Institute (Shenzhen Futian), Nankai University, Tianjin, China

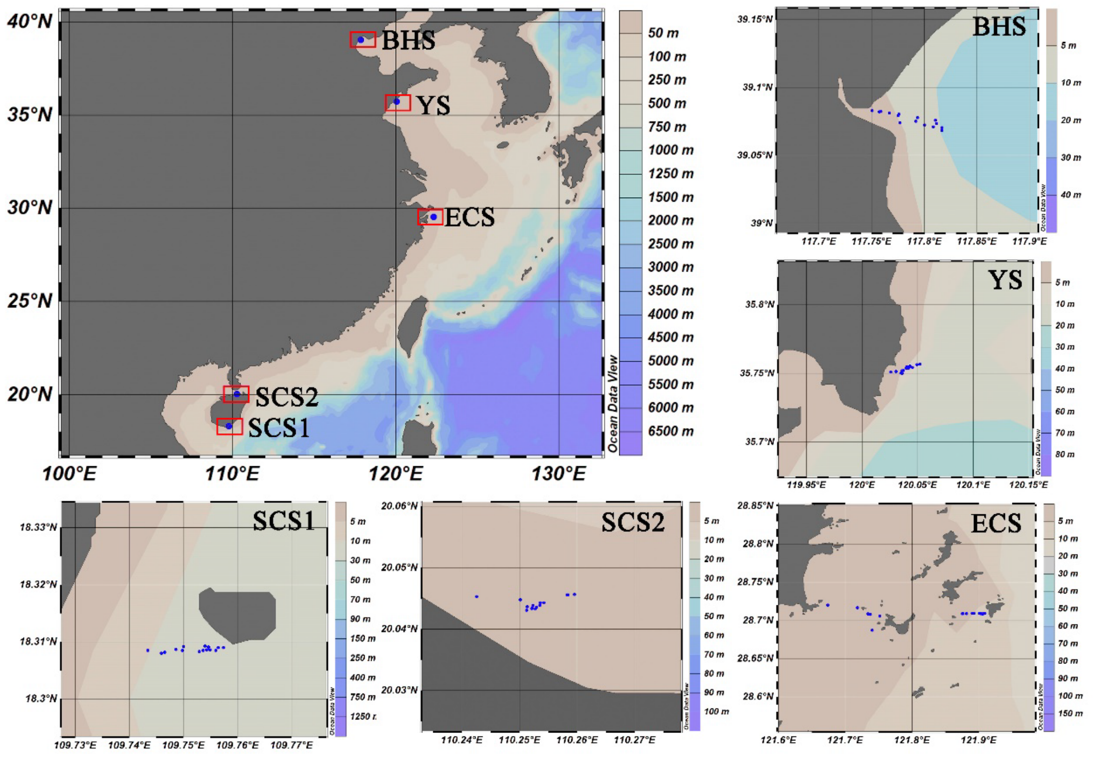

Figure S1 Sampling map in Chinese marginal seas. SCS1, SCS2: South China Sea; BHS: Bohai Sea; YS: Yellow Sea; ECS: East China Sea.

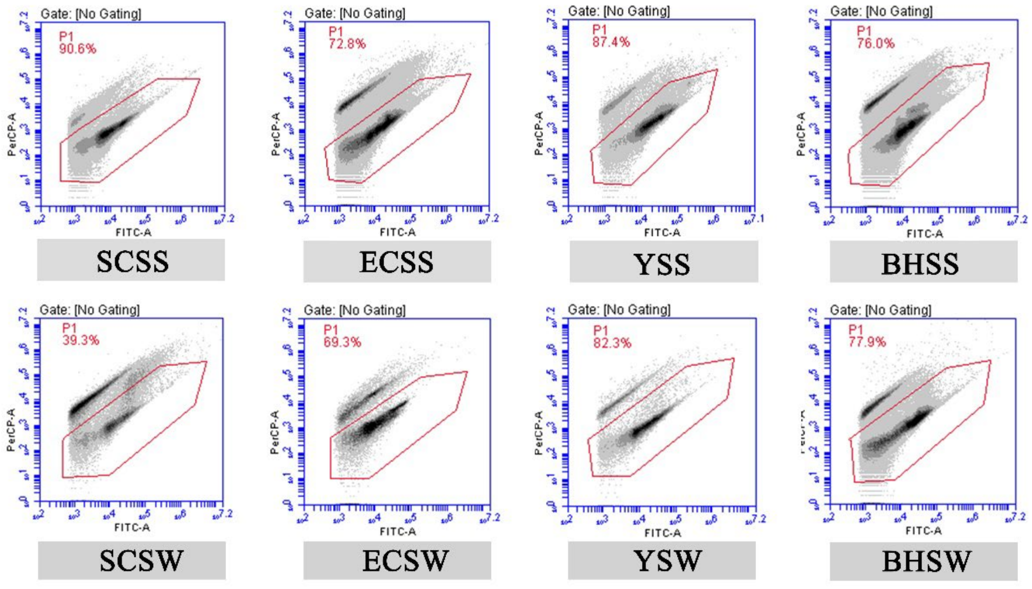

Figure S2 Fixed gate on flow cytometric fingerprint for the samples in this study. All data were obtained by excluding the abiotic background using the fixed gate P1. SCSS: samples from South China Sea in summer; SCSW: samples from South China Sea in winter; ECSS: samples from East China Sea in summer; ECSW: samples from East China Sea in winter; YSS: samples from Yellow Sea in summer; YSW: samples from Yellow Sea in winter; BHSS: samples from Bohai Sea in summer; BHSW: samples from Bohai Sea in winter

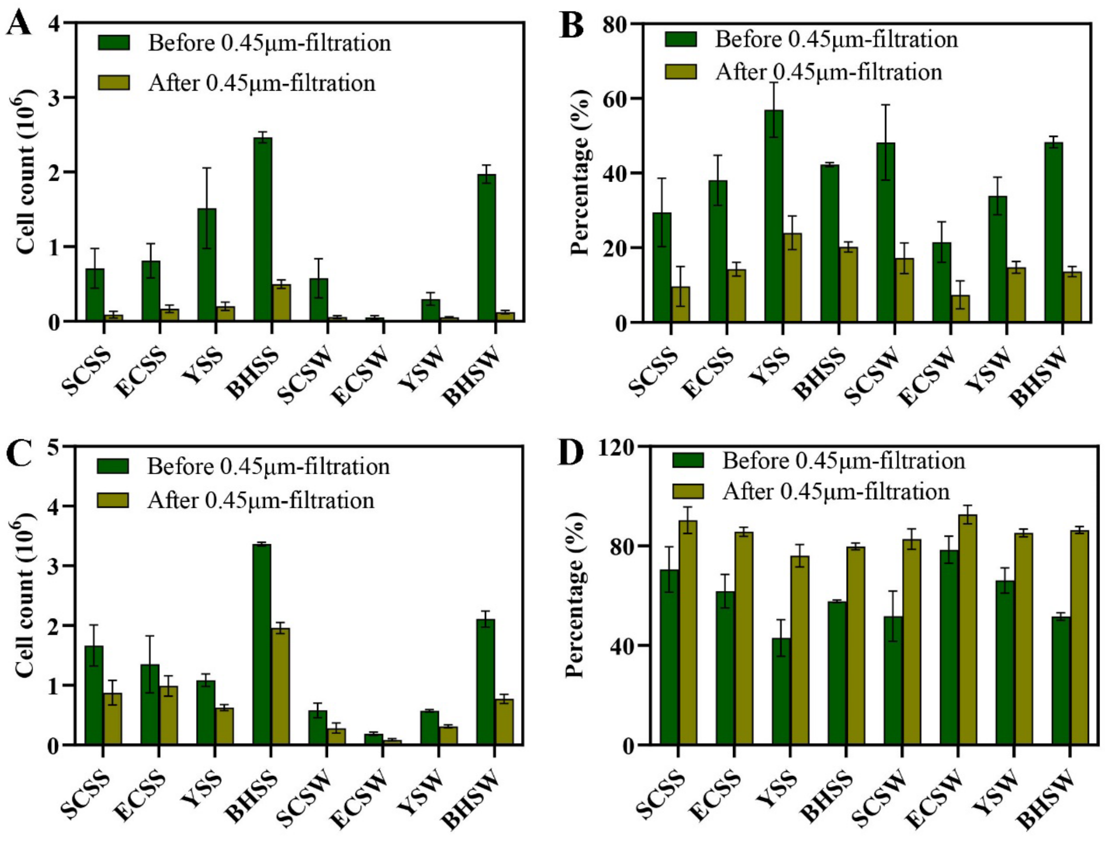

Figure S3 Changes in cell concentration and percentage of HNA and LNA bacteria before and after 0.45μm-filtration from the samples in different sites at two seasons. A, B: HNA bacteria; C, D: LNA bacteria. SCSS: samples from South China Sea in summer; SCSW: samples from South China Sea in winter; ECSS: samples from East China Sea in summer; ECSW: samples from East China Sea in winter; YSS: samples from Yellow Sea in summer; YSW: samples from Yellow Sea in winter; BHSS: samples from Bohai Sea in summer; BHSW: samples from Bohai Sea in winter

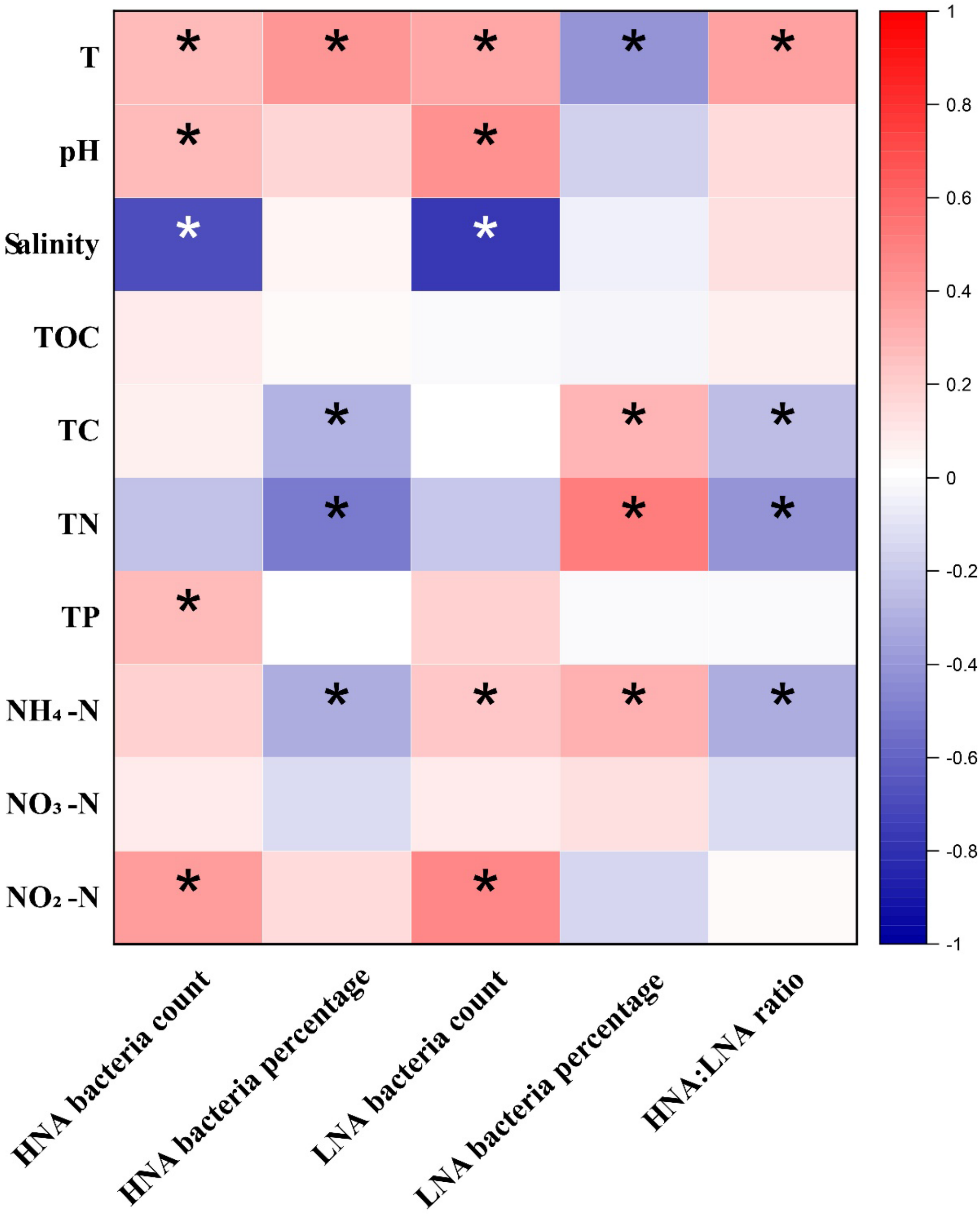

Figure S4 Pearson correlations between the count, percentage of the “HNA” and “LNA” groups, HNA:LNA ratio with the environmental factors of the samples

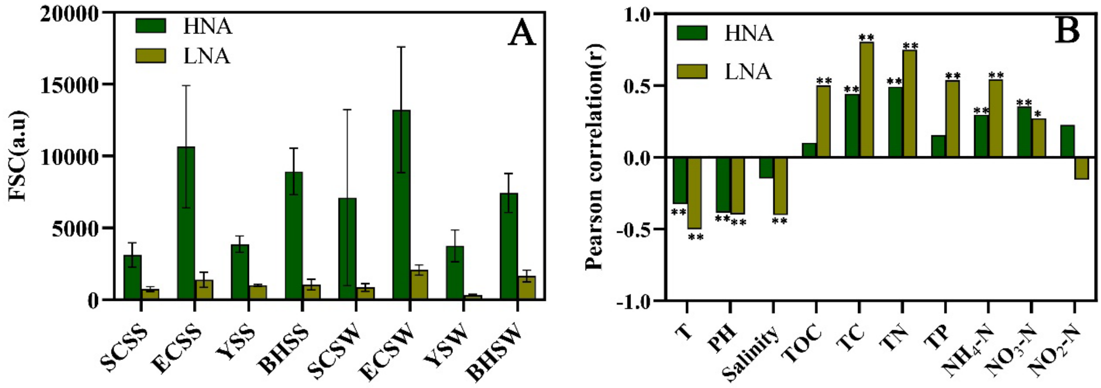

Figure S5 Median value of forward scatter (FSC) light signal (cell size) of "HNA," and "LNA," groups through flow cytometric fingerprint of the samples in different sites at two seasons (A) and Pearson correlations between FSC median value of the "HNA" and "LNA" groups and environmental factors of the samples (B). Labels (* and **) represent significantly ($P < 0.05$ and $P < 0.01$) positive or negative correlations. SCSS: samples from South China Sea in summer; SCSW: samples from South China Sea in winter; ECSS: samples from East China Sea in summer; ECSW: samples from East China Sea in winter; YSS: samples from Yellow Sea in summer; YSW: samples from Yellow Sea in winter; BHSS: samples from Bohai Sea in summer; BHSW: samples from Bohai Sea in winter

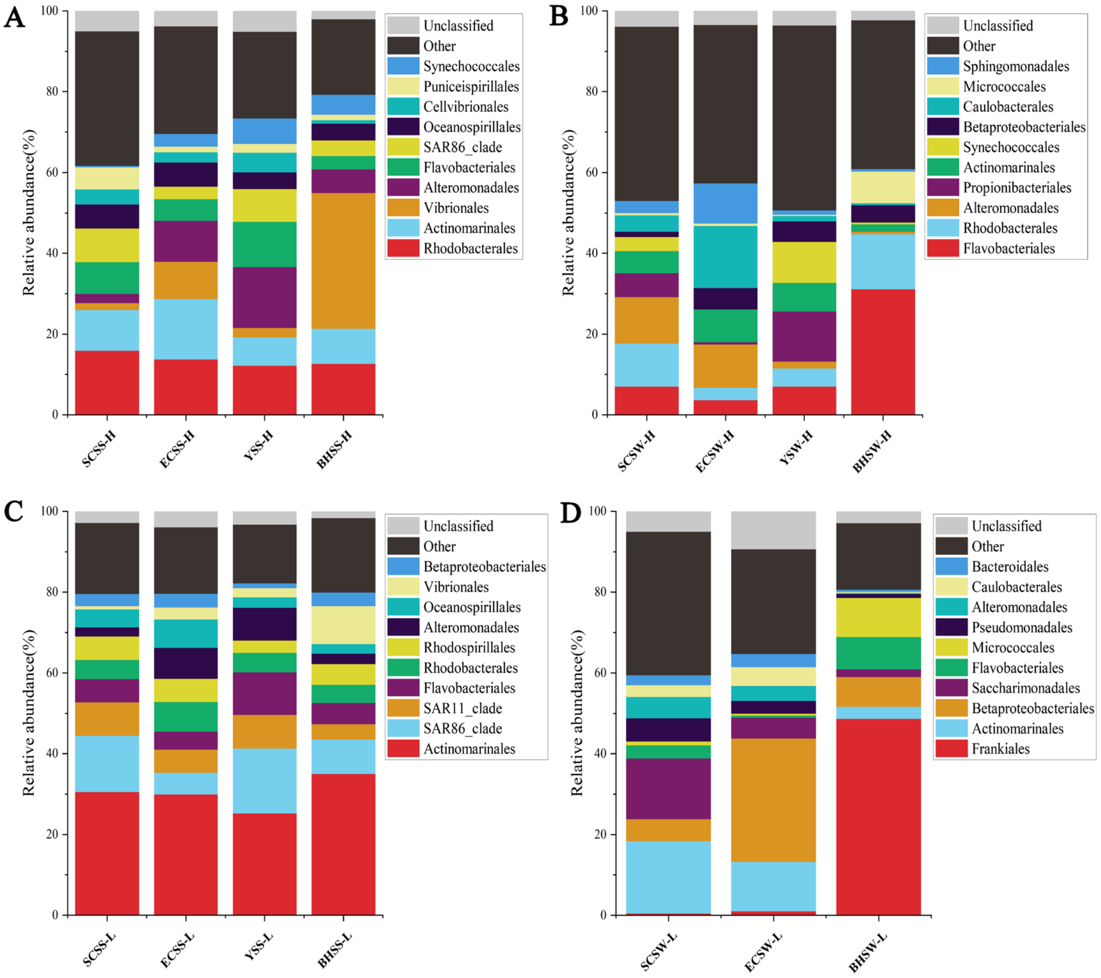

Figure S6 The composition of HNA and LNA bacterial communities at the order level, with the top ten order level chosen in each sample. A: the composition of HNA bacterial community in summer; B: the composition of HNA bacterial community in winter; C: the composition of LNA bacterial community in summer; D: the composition of LNA bacterial community in winter. SCSS: samples from South China Sea in summer; SCSW: samples from South China Sea in winter; ECSS: samples from East China Sea in summer; ECSW: samples from East China Sea in winter; YSS: samples from Yellow Sea in summer; YSW: samples from Yellow Sea in winter; BHSS: samples from Bohai Sea in summer; BHSW: samples from Bohai Sea in winter

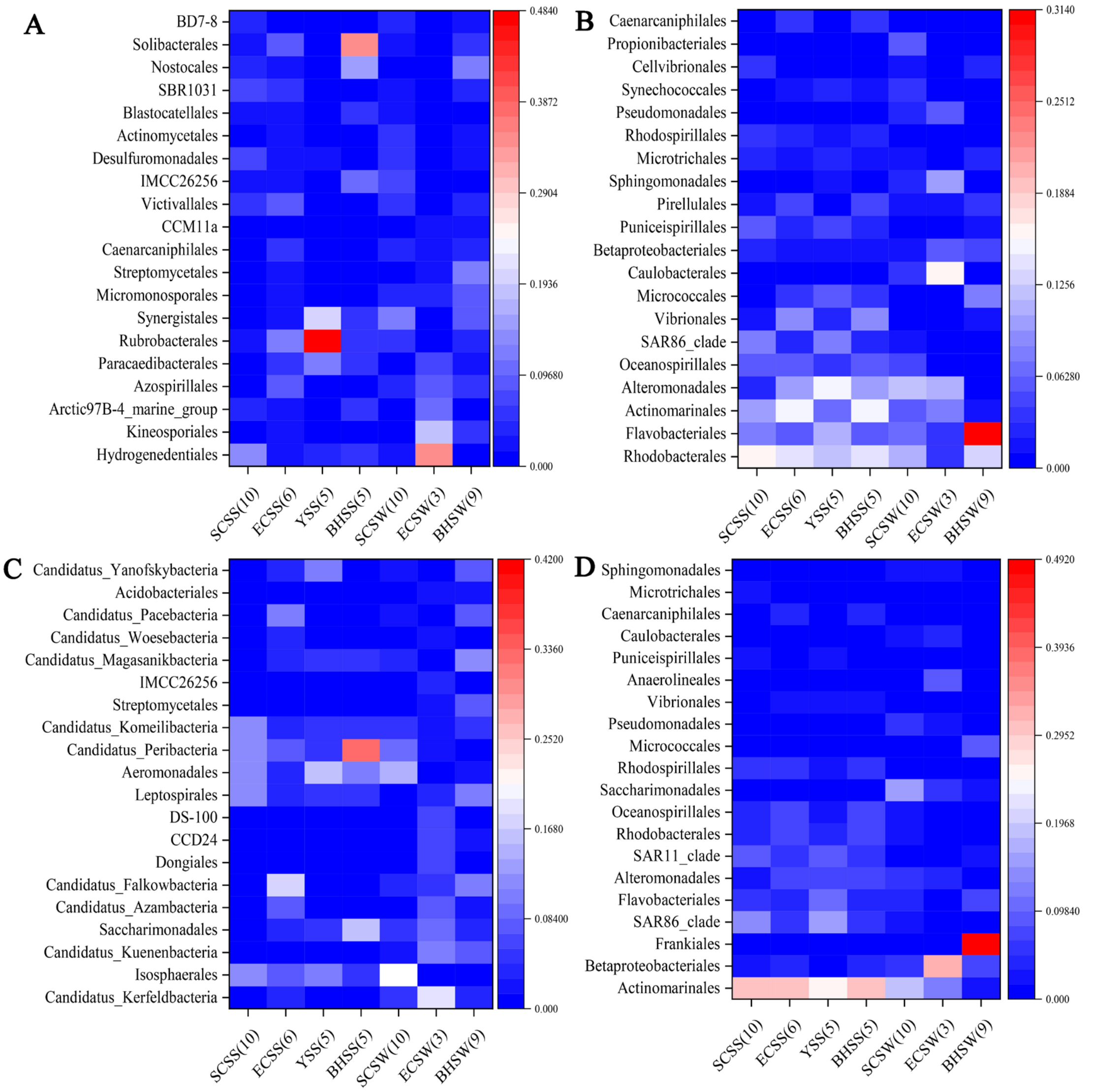

Figure S7 The composition of each phylogenetic group at the order (top 20) levels in each site at different season. Samples from the same area and same season are combined and numbers in parenthesis indicate the number of samples for each group. A: HNA-exclusive bacteria, B: HNA shared with LNA bacteria, C: LNA- exclusive bacteria, D: LNA shared with HNA bacteria. SCSS: samples from South China Sea in summer; SCSW: samples from South China Sea in winter; ECSS: samples from East China Sea in summer; ECSW: samples from East China Sea in winter; YSS: samples from Yellow Sea in summer; YSW: samples from Yellow Sea in winter; BHSS: samples from Bohai Sea in summer; BHSW: samples from Bohai Sea in winter.

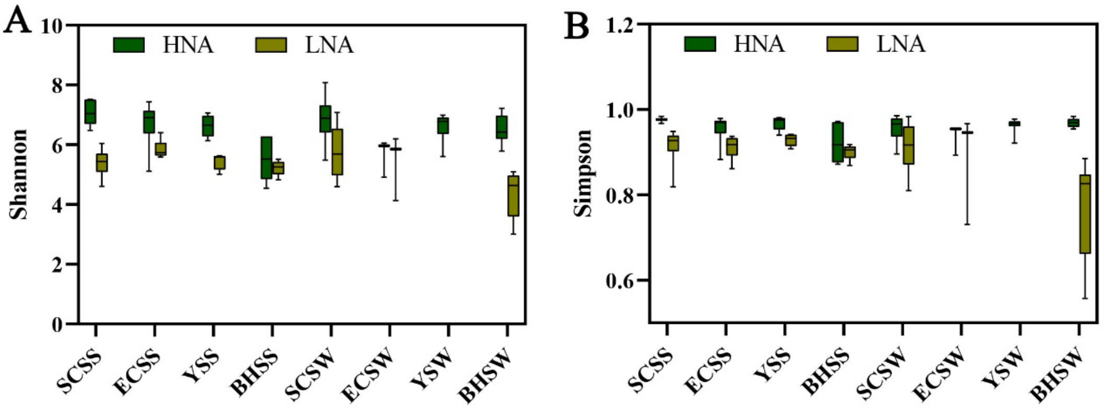

Figure S8 The diversity of HNA and LNA bacteria in Chinese marginal seas. Indices of alpha diversity shown as Shannon (A), Simpson (B). SCSS: samples from South China Sea in summer; SCSW: samples from South China Sea in winter; ECSS: samples from East China Sea in summer; ECSW: samples from East China Sea in winter; YSS: samples from Yellow Sea in summer; YSW: samples from Yellow Sea in winter; BHSS: samples from Bohai Sea in summer; BHSW: samples from Bohai Sea in winter.

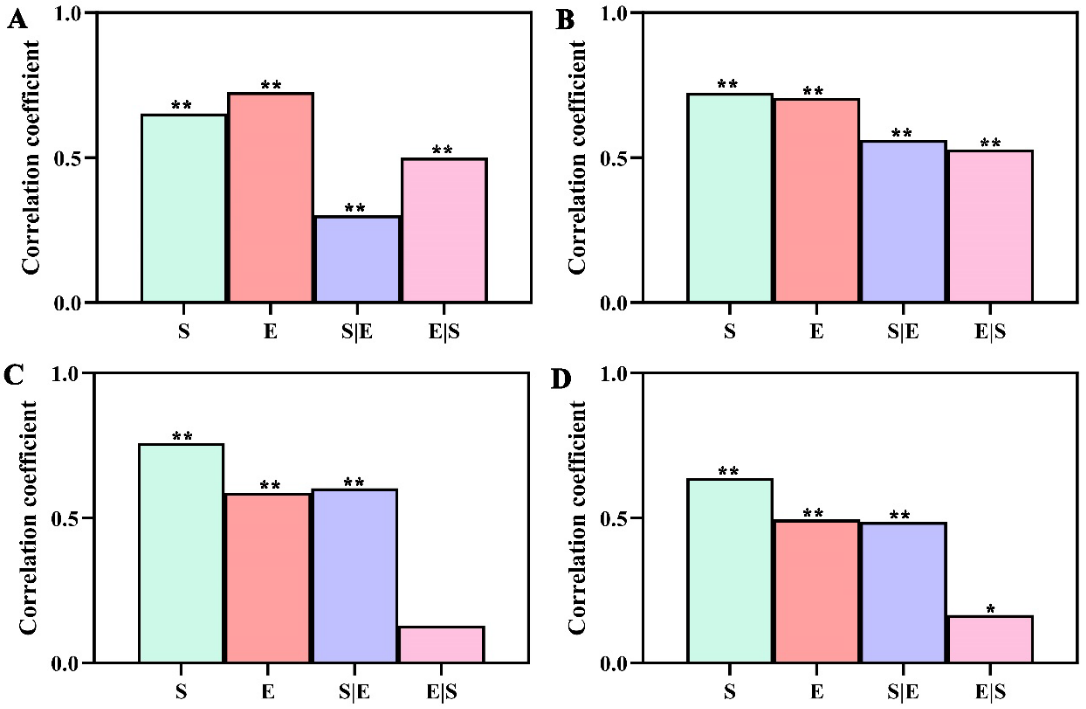

Figure S9 Mantel and partial Mantel tests for the correlation between community dissimilarity of HNA and LNA bacteria and environmental and spatial factors using Pearson's coefficient. *P<0.05, **P<0.01. S|E: pure spatial variation, E|S: pure environmental variation. A: HNA bacteria in summer; B: HNA bacteria in winter; C: LNA bacteria in summer; D: LNA bacteria in winter.

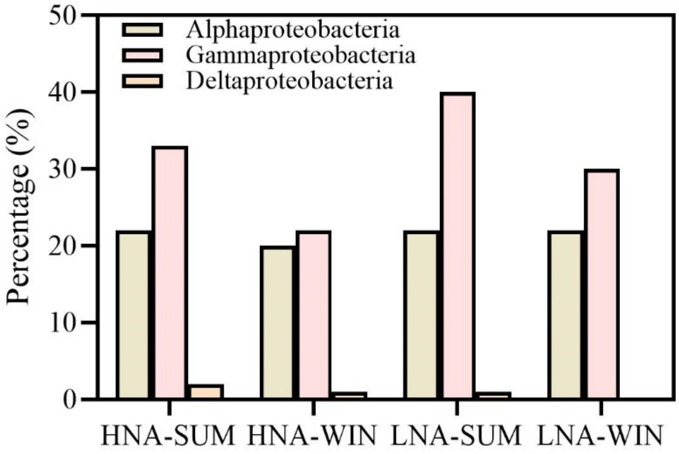

Figure S10 the percentage of Proteobacteria subclasses in the co-occurrence networks. HNA-SUM: HNA bacteria in summer; HNA-WIN: HNA bacteria in winter; LNA-SUM: LNA bacteria in summer; LNA-WIN: LNA bacteria in winter

Table S1 Summary of environmental factors of all samples across summer and winter seasons.

Sample	T(°C)	PH	Salinity/‰	TOC（mg/l)	TC(mg/l)	TN(mg/l)	TP(mg/l)	NH_4-N(mg/l)	NO_3-N(mg/l)	NO_2-N(mg/l)	longitude	latitude
SCSS1	29.1	8.02	29	1.76	22.98	0.1165	0.15	0.05	0.04	0.024	109.75	18.3092
SCSS2	29.2	8.05	30	1.35	22.36	0.1203	0.15	0.02	0.04	0.022	109.7546	18.3091
SCSS3	29.1	8.03	30	1.27	22.29	0.08587	0.19	0.06	0.03	0.023	109.754	18.3093
SCSS4	29.2	8.06	30	1.44	22.72	0.1329	0.13	0.09	0.04	0.015	109.7564	18.309
SCSS5	29.2	8.09	29	1.26	22.26	0.1906	0.16	0.07	0.04	0.01	109.7574	18.309
SCSS6	28.8	8.1	30	1.53	22.82	0.5299	0.09	0	0	0.009	110.2536	20.0443
SCSS7	28.8	8.18	30	1.6	23.04	0.5062	0.03	0.02	0.01	0.005	110.2527	20.0433
SCSS8	29.1	8.07	31	1.79	22.91	0.4105	0.03	0	0.01	0.003	110.2513	20.0437
SCSS9	29.1	8.12	30	1.16	23.18	0.5037	0.07	0.01	0	0.006	110.2595	20.0457
SCSS10	29.1	8.15	30	1.54	22.53	0.3813	0.08	0.02	0	0.002	110.2582	20.0456
ECSS1	28.4	7.83	27	2.63	39.96	0.6031	0.25	0.01	0.04	0.022	121.7399	28.6873
ECSS2	28.4	7.89	27	1.38	22.63	0.3696	0.23	0	0.04	0.023	121.7514	28.7059
ECSS3	28.3	7.85	27	1.25	22.63	0.298	0.26	0.01	0.05	0.023	121.7375	28.7081
ECSS4	28.4	7.81	27	1.55	23.48	0.3429	0.3	0.01	0.05	0.028	121.7341	28.7089
ECSS5	28.3	7.85	27	0.954	22.25	0.2972	0.33	0	0.04	0.022	121.718	28.7169
ECSS6	28.4	7.88	27	1.07	22.75	0.3397	0.3	0	0.05	0.026	121.6737	28.7204
YSS1	29.3	7.97	32	1.57	23.8	0.295	0.17	0	0.01	0.005	120.043	35.7552
YSS2	29.3	8.03	32	1.02	23.39	0.2305	0.1	0	0.01	0.006	120.0396	35.7539
YSS3	29.3	8.01	32	1.93	23.91	0.2366	0.14	0.01	0.01	0.006	120.036	35.7519
YSS4	29.3	7.99	32	1.7	23.41	0.2156	0.15	0	0.01	0.006	120.04	35.7548
YSS5	29.4	8.01	32	1.78	23.79	0.2556	0.14	0	0.01	0.004	120.0405	35.755
BHSS1	28.1	7.92	25	5.51	30.4	0.6577	0.09	0.07	0.03	0.023	117.7572	39.0822
BHSS2	28.1	7.98	25	3.23	27.05	0.3794	0.13	0.04	0.02	0.026	117.7757	39.0804
BHSS3	28.2	7.98	25	3.05	27.98	0.6552	0.08	0.07	0.03	0.025	117.7934	39.078
BHSS4	28.1	7.95	25	3.43	27.91	0.3931	0.08	0.05	0.02	0.022	117.8105	39.076
BHSS5	28.1	7.95	25	5.07	28.86	0.5653	0.09	0.05	0.03	0.022	117.8113	39.0736
SCSW1	25.7	7.794	34	4.45	26.05	0.3054	0.09	0	0.01	0.003	109.7434	18.3085
SCSW2	25.5	7.855	34	3.39	24.23	0.2484	0.1	0	0	0.003	109.7459	18.308
SCSW3	25.5	7.827	34	3.53	24.37	0.2802	0.18	0	0	0.002	109.7486	18.3087
SCSW4	25.7	8.011	34	4.12	23.68	0.3176	0.09	0.01	0	0.003	109.7465	18.3082
SCSW5	25.6	7.794	33	4.57	26.2	0.4681	0.14	0	0	0.001	109.7498	18.3085
SCSW6	25.6	7.799	34	12.83	32.38	0.206	0.06	0	0.01	0.002	109.7529	18.3083
SCSW7	25.7	8.002	34	3.57	23.71	0.213	0.08	0	0	0.002	109.7543	18.3086
SCSW8	25.7	7.787	33	4.66	26.21	0.3879	0.05	0.01	0	0.002	109.7536	18.3085
SCSW9	25.6	7.998	34	4.62	24.05	0.2513	0.08	0	0	0.002	109.7549	18.3086
SCSW10	25.5	7.785	34	4.26	24.33	0.2563	0.09	0	0.01	0.002	109.756	18.3085
SCSW11	26.4	7.87	34	1.13	22.5	0.2803	0.04	0	0.02	0.018	110.2542	20.0443
SCSW12	26.3	7.882	34	1.14	22.68	0.2744	0.07	0	0.03	0.019	110.2535	20.0439
SCSW13	26.4	7.873	34	1.3	22.87	0.28	0.07	0	0.03	0.017	110.2529	20.0436
SCSW14	26.1	7.883	33	1.18	22.26	0.2279	0.04	0	0.04	0.023	110.2523	20.0439
SCSW15	26.3	7.882	34	1.13	22.52	0.25	0.04	0	0.04	0.024	110.2523	20.0438
SCSW16	26.1	7.865	34	1.13	22.43	0.2687	0.03	0.01	0.03	0.022	110.2521	20.0434

SCSW17	26.2	7.855	34	0.802	22.13	0.2278	0.06	0.01	0.04	0.022	110.2512	20.0431
SCSW18	26.4	7.861	33	1.135	22.83	0.2672	0.09	0	0.04	0.02	110.2501	20.0448
SCSW19	26.4	7.858	34	0.917	22.41	0.2361	0.07	0	0.03	0.027	110.2425	20.0453
SCSW20	26.3	7.859	34	0.969	22.55	0.2265	0.05	0	0.02	0.019	110.2584	20.0456
ECSW1	9	7.663	30	5.13	37.71	2.1321	0.49	0.05	0.08	0.003	121.8752	28.7092
ECSW2	9.1	7.727	30	6.81	41.67	2.6061	0.22	0.05	0	0.003	121.876	28.7093
ECSW3	9.6	7.75	31	4.29	39	2.3835	0.09	0.1	0.01	0.005	121.8898	28.7096
ECSW4	9	7.781	30	3.69	38.76	1.9938	0.16	0.07	0	0.004	121.8923	28.7094
ECSW5	9.6	7.715	30	4.83	41.46	1.9791	0.14	0.08	0.08	0.003	121.9048	28.7089
ECSW6	9.8	7.641	31	4.68	40.08	1.9989	0.14	0.07	0.03	0.002	121.9069	28.7091
ECSW7	9.2	7.559	31	4.74	40.05	1.938	0.11	0.07	0.01	0.007	121.9025	28.7092
ECSW8	9.3	7.558	30	4.41	36.75	2.1357	0.1	0.07	0.01	0.002	121.9087	28.7094
ECSW9	9.7	7.783	30	4.44	37.5	1.9584	0.09	0.11	0.13	0.008	121.9004	28.7095
ECSW10	9.5	7.518	31	4.32	39.45	1.9659	0.09	0.09	0.04	0.005	121.8812	28.7091
YSW1	17.9	7.686	32	1.39	23.8	0.1944	0.14	0	0.01	0.008	120.0257	35.7511
YSW2	18	7.771	32	1.29	23.79	0.1784	0.14	0	0.01	0.007	120.035	35.7503
YSW3	17.6	7.774	32	1.54	23.86	0.1615	0.12	0.01	0.01	0.008	120.0359	35.75
YSW4	17.3	7.752	32	1.65	23.77	0.2083	0.13	0	0.02	0.011	120.0453	35.7544
YSW5	17.5	7.753	32	1.4	23.81	0.1865	0.12	0.01	0.01	0.009	120.037	35.7521
YSW6	17.4	7.759	32	1.6	24.32	0.1968	0.08	0	0.02	0.01	120.0304	35.7515
YSW7	17.5	7.755	32	1.4	23.93	0.1811	0.1	0	0.01	0.01	120.04133	35.7537
YSW8	17.8	7.751	32	1.85	24.4	0.2346	0.05	0	0.01	0.009	120.0432	35.7553
YSW9	17.9	7.749	32	1.72	24.27	0.1919	0.11	0	0.01	0.007	120.0493	35.7564
YSW10	17.2	7.749	32	1.82	24.22	0.1811	0.12	0	0.01	0.008	120.052	35.7567
BHSW1	16.7	7.834	26	3.95	36.02	0.9792	0.45	0.05	0.02	0.006	117.8166	39.0684
BHSW2	16.6	7.904	26	4.04	35.35	0.8208	0.39	0.12	0.02	0.006	117.8165	39.0706
BHSW3	16.7	7.806	26	3.45	33.22	0.6805	0.27	0.02	0.04	0.011	117.8084	39.0711
BHSW4	16.8	7.775	26	3.61	33.68	0.7027	0.2	0.02	0.03	0.012	117.8001	39.0724
BHSW5	16.9	7.782	26	3.9	33.83	0.7354	0.44	0.03	0.04	0.014	117.7917	39.0753
BHSW6	16.5	7.814	25	4.1	34.95	0.7561	0.29	0.03	0.05	0.017	117.775	39.0796
BHSW7	16.6	7.723	25	3.86	33.82	0.6342	0.28	0.06	0.04	0.014	117.7667	39.0814
BHSW8	16.7	7.749	25	3.71	33.11	0.5863	0.19	0.05	0.03	0.014	117.7585	39.0826
BHSW9	16.3	7.702	24	4.04	33.25	0.6461	0.13	0.05	0.03	0.018	117.7502	39.0832
BHSW10	16.6	7.689	24	3.91	35.13	0.7998	0.18	0.12	0.05	0.017	117.7768	39.0741

Table S2 Relative importance of various factors (space, season, and interactions between these two factors) in the cell concentration percentage and FSC value of HNA and LNA bacterial communities, calculated by Person analysis.

		Spatial		Season		Spatial×Season	
		F-value	*p*-value	*F*-value	*p*-value	*F*-value	*p*-value
HNA	Cell concentration	177.103	0.000	125.999	0.000	17.835	0.000
	Cell percentage	14.468	0.000	4.042	0.048	32.577	0.000
	FSC	13.627	0.000	1.613	0.208	1.836	0.149
	Cell concentration	296.691	0.000	397.459	0.000	9.564	0.000
LNA	Cell percentage	14.419	0.000	4.099	0.047	32.741	0.000
	FSC	41.882	0.000	5.688	0.020	14.572	0.000

Table S3 Average number and percent of exclusive and shared OTUs within HNA and LNA bacteria among the samples.

Sample	HNA		LNA	
	Exclusive	Shared	Exclusive	Shared
SCSS(n=10)	973(54%)	833(46%)	378(31%)	833(69%)
ECSS(n=6)	699(45%)	870(55%)	514(37%)	870(63%)
YSS(n=5)	591(49%)	611(51%)	370(38%)	611(62%)
BHSS(n=5)	432(41%)	634(59%)	438(41%)	634(59%)
SCSW(n=10)	1291(74%)	465(26%)	289(38%)	465(62%)
ECSW(n=3)	272(56%)	218(44%)	269(55%)	218(45%)
BHSW(n=9)	566(59%)	389(41%)	328(46%)	389(54%)

Table S4 the definition of each cluster

Cluster	Definition
Cluster1	The genera shared between HEW (exclusive HNA bacteria in winter) and HES (exclusive HNA bacteria in summer)
Cluster2	The genera shared between HES (exclusive HNA bacteria in summer) and HLSW (shared between HNA and LNA bacteria in winter)
Cluster3	The genera shared between HES (exclusive HNA bacteria in summer) and LEW (exclusive LNA bacteria in winter)
Cluster4	The exclusive genera in HES (exclusive HNA bacteria in summer)
Cluster5	The genera shared between HEW (exclusive HNA bacteria in winter) and HLSS (shared between HNA and LNA bacteria in summer)
Cluster6	The genera shared between HEW (exclusive HNA bacteria in winter) and LES (exclusive LNA bacteria in summer)
Cluster7	The exclusive genus in HEW (exclusive HNA bacteria in winter)
Cluster8	The genera shared between HLSS (shared between HNA and LNA bacteria in summer) and HLSW (shared between HNA and LNA bacteria in winter)
Cluster9	The genera shared between LES (exclusive LNA bacteria in summer) and HLSW (shared between HNA and LNA bacteria in winter)
Cluster10	The exclusive genus in HLSW (shared between HNA and LNA bacteria in winter)
Cluster11	The genera shared between HLSS (shared between HNA and LNA bacteria in summer) and LEW (exclusive LNA bacteria in winter)
Cluster12	The exclusive genera in HLSS (shared between HNA and LNA bacteria in summer)
Cluster13	The exclusive genus in LES (exclusive LNA bacteria in summer)
Cluster14	The exclusive genus in LEW (exclusive LNA bacteria in winter)
Cluster15	The genera shared between LES (exclusive LNA bacteria in summer) and LEW (exclusive LNA bacteria in winter)

Table S5 Relative importance of various factors (space, season, and interactions between these two factors) in the diversity of HNA and LNA bacterial communities, calculated by Person analysis.

		Spatial		Season		Spatial×Season	
		F-value	*p*-value	*F*-value	*p*-value	*F*-value	*p*-value
HNA	Shannon	7.795	0.000	0.242	0.625	5.360	0.003
	Simpson	2.955	0.042	0.002	0.965	5.176	0.004
LNA	Shannon	4.696	0.007	2.561	0.117	3.637	0.035
	Simpson	3.819	0.017	6.183	0.017	4.106	0.024

Table S6 Community comparison based on the PERMANOVA analysis

diffs	*F*-value	R^2	*P*-value	significant
HNA-SUMMER **vs** HNA-WINTER	6.843	0.1143	0.001	**
LNA-SUMMER **vs** LNA-WINTER	10.6015	0.1873	0.001	**
HNA-SUMMER **vs** LNA-SUMMER	2.3391	0.0447	0.002	**
HNA-WINTER **vs** LNA-WINTER	3.4758	0.0662	0.001	**
BHSS-HNA **vs** ECSS-HNA **vs** YSS-HNA **vs** SCSS-HNA	3.605	0.3296	0.001	**
BHSS-LNA **vs** ECSS-LNA **vs** YSS-LNA **vs** SCSS-LNA	3.2061	0.3042	0.001	**
BHSW-HNA **vs** ECSW-HNA **vs** YSW-HNA **vs** SCSW-HNA	4.2935	0.34	0.001	**
BHSW-LNA **vs** ECSW-LNA **vs** SCSW-LNA	2.0629	0.1784	0.001	**

Table S7 Topological parameters of the interaction networks of HNA and LNA bacteria

Parameter	HNA-SUMMER	HNA-WINTER	LNA-SUMMER	LNA-WINTER
Nodes	224	207	166	149
links	3645	5244	1880	1466
Positive links (%)	70.29	86.94	62.23	93.39
Average weighted degree	19.549	56.089	8.232	26.227
